# Dual Aspect of the Pandemic on the African Continent: Viral Distribution and Shifting Demographic Susceptibility to SARS-CoV-2

**DOI:** 10.3390/v18050524

**Published:** 2026-04-30

**Authors:** Julia Cyrielle Andeko, Sonia Etenna Lekana-Douki, Gabriel Falque, Nadine N’dilimabaka, Jean-Bernard Lekana-Douki

**Affiliations:** 1 Centre Interdisciplinaire de Recherches Médicales de Franceville (CIRMF), Franceville BP 769, Gabon; juliatesse@gmail.com (J.C.A.); gabfalque@hotmail.fr (G.F.); nadinendilimabaka@yahoo.fr (N.N.); lekana_jb@yahoo.fr (J.-B.L.-D.); 2 Département de Biologie, Faculté des Sciences, Université des Sciences et Techniques de Masuku (USTM), Franceville BP 901, Gabon; 3 Ecole des Sciences et Medecine Vétérinaire de Masuku (ESMVM), Université des Sciences et Techniques de Masuku (USTM), Franceville BP 901, Gabon; 4 Département de Parasitologie-Mycologie, Université des Sciences de la Santé (USS), Libreville BP 4009, Gabon

**Keywords:** SARS-CoV-2, genomic surveillance, variants, viral evolution, circulation in Africa

## Abstract

SARS-CoV-2, the causative agent of COVID-19, emerged in late 2019 and rapidly developed into a global health crisis. In this study, we analysed 173,194 SARS-CoV-2 genomes from the GISAID database to explore the intra-continental dynamics and distribution of variants across Africa between 2020 and 2024. We have identified 1377 distinct lineages, which were classified by clade to assess associations with infection and mortality rate. So, we conducted a Shannon entropy analysis to confirm the diversity and we applied a Correspondence Analysis (CA). Our findings revealed that one of the deadliest in Africa during the Delta wave, lineage AY.45 predominated in the South Africa cluster, whereas AY.34.1 drove transmission in the Atlantic West Africa cluster, underscoring regional heterogeneity. Furthermore, early in the pandemic, men exhibited a 39% higher risk of infection compared to women (aOR: 1.39, 95% CI [1.34–1.45]), particularly in association with clade G. By contrast, later stages were dominated by clade GRA, which disproportionately affected the elderly (≥70 years; aOR: 1.39, 95% CI [1.33–1.45]) and children (0–9 years; aOR: 1.26, 95% CI [1.20–1.33]). Our analysis highlighted that the pandemic on the African continent unfolded as a mosaic of epidemics shaped by diverse variants and regional epidemiological contexts. These findings emphasize the importance of genomic surveillance to capture local epidemic signatures and inform region-specific public health strategies.

## 1. Introduction

Since its emergence in late 2019, SARS-CoV-2, the causative agent of the COVID-19 pandemic, has profoundly disrupted global public health, resulting in more than 676 million cases and 6.88 million deaths worldwide by 2024 [1,2]. This virus, belonging to the *Coronaviridae* family, is characterized by a high mutation rate (1.1 × 10^−3^ per site per year), corresponding to approximately 31.5 nucleotide substitutions per genome [3,4]. Such genomic plasticity has driven the emergence of new variants, responsible for successive epidemic waves [1].

Mutations predominantly affect the Spike protein, particularly the N-terminal domain (NTD) and the receptor-binding domain (RBD), which determine affinity for the hACE2 receptor and promote immune evasion [5]. These molecular changes have led to the classification of viral lineages into Variants of Interest (VOIs) and Variants of Concern (VOCs), such as Alpha, Beta, Delta, and Omicron, which are distinguished by increased transmissibility, immune escape capacity, and clinical impact [6,7]. Omicron and its sublineages currently dominate global circulation, including across Africa [8].

From a phylogenetic perspective, SARS-CoV-2 has been classified into eleven clades (G, S, L, V, O, GR, GK, GV, GH, GRA, and GRY), reflecting its genetic diversity and evolutionary dynamics [9,10,11]. Genomic sequencing therefore represents an essential tool for the early detection of emerging variants, tracking their geographic spread, and assessing the impact of mutations on infectivity, disease severity, and immune escape, thereby informing vaccine updates and guiding public health strategies [12].

However, the implementation of genomic surveillance systems remains uneven. While high-income countries have consolidated their sequencing capacities, many low- and middle-income countries, particularly in Africa, continue to face major challenges related to laboratory infrastructure, financial resources, and logistical barriers [13]. Initiatives led by the Africa CDC and the African Union have contributed to strengthening sequencing capacity [14]. Nevertheless, large regions of the continent remain underrepresented and countries such as South Africa, Nigeria, and Kenya have made notable contributions to global databases [15]. This underrepresentation limits the understanding of regional variant circulation dynamics and delays the development of timely public health responses [15,16]. In addition, important knowledge gaps persist regarding the impact of variants on immunity, reinfection risk, vaccine effectiveness, and clinical severity stratified by age, sex, and comorbidities within African populations [7,14].

Continent-wide studies have described the spatial and temporal prevalence of SARS-CoV-2 variants in Africa [16,17]. Subsequent work revealed that the continental pandemic was not a uniform process but rather a dynamic mosaic, with specific variants and lineages shaping distinct regional epidemics before the more widespread circulation of Delta and Omicron [18]. Separately, national studies in countries like Cameroon and Nigeria connected specific variants to local mortality trends [19,20]. Finally, changes in demographic tropism (i.e., variation in the age and sex profiles targeted by successive variants) have been well documented in Europe and North America [21].

However, these different approaches have limitations. A continent-wide view risks masking important regional dynamics. At the same time, national studies cannot follow the cross-border transmission routes that connect different countries. Finally, a systematic analysis of how risk profiles evolve within different African populations is lacking.

Our study was designed to combine these different scales of analysis. Rather than providing a simple description, we propose a robust framework based on the assessment of epidemiological basins. We used this framework to (i) deconstruct the continental waves into their regional components, (ii) understand the severity of the variants through complementary analyses of case fatality rates (CFR) and their relative impact (multivariate regression), and (iii) systematically characterise the three successive demographic profiles that defined the pandemic in Africa.

## 2. Material and Methods

### 2.1. Data Collection

On 5 December 2024, we collected 173,194 SARS-CoV-2 genome sequences obtained from the GISAID database originating from the African continent [22]. To ensure the quality and robustness of the analyses, we cleaned the data obtained by removing sequences that did not conform to the dd-mm-yyyy collection date format, lacked lineage assignment or originated from non-human hosts. For demographic risk analysis we also removed data missing host age information. Key metadata fields retained in the dataset included GISAID accession number, country of origin, collection date, lineage (assigned using Pango lineage [9,23]), Nextstrain clade classification, host species, sex, age, vaccination status, and amino acid substitutions within genome. Daily epidemiological data for each country concerning the number of new cases and death as well as vaccination data were obtained from the Our World in Data directory, which has collected its data from Johns Hopkins University [24].

After filtering 173,148 sequences were retained for cluster-based analysis (geo-temporal distribution of these sequences details in Table S1). For demographic risk analysis we retained 149,148 sequences. The final dataset included sequences from 78,094 females (52.36%) and 71,054 males (47.64%).

Participants with available age data were categorized into six age groups: children (<10 years; 4.07%), teenagers (10–17 years; 6.78%), young adults (18–29 years; 20.42%), adults (30–49 years; 39.12%), older adults (50–69 years; 22.29%), and elderly (≥70 years; 7.33%). The dataset included sequences from 11 clades: GRA (76,175; 51.07%), GK (33,025; 22.14%), GH (15,245; 10.22%), G (9983; 6.69%), GR (9465; 6.35%), GRY (3054; 2.05%), S (1048; 0.70%), O (609; 0.41%), GV (444; 0.30%), L (78; 0.05%), and V (22; 0.01%).

### 2.2. Statistical Analysis

The remained data after cleaning were representing samples collected between 1 January 2020, and 26 November 2024, from 55 African countries. These samples encompassed 1377 unique lineages grouped into 11 clades. Analyses were conducted using Python 3 [25] within the Jupyter Notebook (v7.4.3) environment [26]. Data processing utilized the Pandas (v2.2.3) [27] and NumPy (v2.2.6) [28] libraries, while statistical analyses were performed with SciPy (v1.13.1) and Stats Models (v0.14.2). Data visualizations were generated using Matplotlib (v3.9.0) and Seaborn (v0.13.2).

#### 2.2.1. Defining the Analytical Framework: Spatiotemporal Clustering

In order to establish a robust analytical framework for our ecological analyses, we first identified regions sharing similar patterns of viral circulation. Our analysis entirely focused on the pre-Omicron period (2020–2021). This time frame was explicitly chosen to represent the moment of maximum viral heterogeneity before the global landscape was homogenised by the Omicron variant, confirmed by a Shannon entropy (Figures S1 and S2) [29].

We then applied Correspondence Analysis (CA), a dimension reduction technique for contingency tables, using the prince library. The input was a country-by-lineage matrix built from the 102,735 valid sequences recorded during the pre-Omicron period. The selection of the number of lineages (*N* = 30) and clusters (k = 7) was validated through a systematic sensitivity analysis (Figure S3). This analysis confirmed that (*N* = 30, k = 7) represents the optimal, most parsimonious choice that reveals a clear and stable structure in the data, with other tested parameters resulting in either incomplete or less distinct cluster patterns (country distribution across varying parameters detailed in Table S2) [30].

The first 5 components of this definitive CA, which explained over 62% of the total variance, were then used for clustering. Finally, countries were assigned to one of the seven clusters using the K-Means algorithm implemented in the scikit-learn library (v1.6.1) [24]. This process gave us 7 geographically and genetically coherent regions, which we then used as the basis for all the ecological and epidemiological comparisons (CA results detailed in Figure S1).

#### 2.2.2. Epidemiological and Ecological Analyses

To quantify the association between viral evolution and epidemic dynamics, we calculated the correlations between the prevalence of a variant and the waves of cases and deaths (comprehensive monthly epidemiological data detailed in Table S3). This analysis was carried out for each of our 7 clusters using data aggregated by month between January 2020 and December 2024.

We selected Spearman’s rank correlation (ρ), a non-parametric approach, to ensure the robustness of the analysis. Assuming that an increase in the prevalence of a variant does not necessarily result in an immediate epidemic impact, the application of a correlation without a time lag would be inappropriate. We therefore systematically tested the correlation for time lags of 0; 1 and 2 months. For each variant (lineage or clade), we selected the time lag for with the highest statistically significantly (*p* < 0.05) coefficient.

To strengthen the robustness of our analysis, we also quantified the uncertainty in our correlation coefficients. We calculated a 95% confidence interval for each ρ value using a non-parametric bootstrap procedure. This involved generating 1000 bootstrap replicates by resampling the paired monthly data with replacement. The confidence interval was then defined by the 2.5th and 97.5th percentiles of the distribution of the coefficients thus obtained. All calculations were performed using the SciPy library (v1.13.1) [31].

To estimate the intraspecific severity of a clade, we calculated the time-lagged Case Fatality Rate (CFR). A clade was considered dominant if its prevalence exceeded a threshold of 60% in a given month and cluster. This threshold was selected to ensure a clear signal from a single variant, and this choice was validated through a sensitivity analysis in which all calculations were repeated for thresholds of 50% and 75%. This confirmed the conclusion that the hierarchy of relative severity of variants was not dependent on these specific values. To account for the delay between the case reporting and the death occurrence, the CFR for each period of dominance was calculated as the ratio of the total number of deaths in the subsequent month (M + 1) to the number of cases reported during the month of dominance (M). This one-month lag is a methodological approximation that considers clinical estimates of the median time between symptom onset and death (approx. 13–22 days) [26,32]. For each CFR value obtained in this way, a 95% confidence interval was calculated using the Wilson score method [33]. Finally, to determine whether the observed differences in CFR between variants within the same cluster were statistically significant, we performed a Pearson’s chi-squared test on the contingency table of the outcomes (death or survival) for cases associated with each dominant clade.

To quantify the relative impact of each viral variant and vaccination on mortality, we applied a separate multivariate Ridge regression model to each cluster using scikit-learn. The target variable was the total number of deaths per month. The predictor variables included the monthly proportion of circulating clades and the monthly average vaccination rate (people fully vaccinated per hundred). To ensure model stability and avoid spurious associations from low-prevalence variants, we applied a double threshold filter prior to regression for each cluster. Only clades reaching a minimum peak prevalence of 3% in at least one month and maintaining an average monthly prevalence of at least 1% were included as predictors for the cluster-specific model. This approach limits the influence of individual data points that could have a disproportionate effect on regression coefficients. The analysis thus remains focused on clades with a strong epidemiological footprint within each cluster.

Ridge regression was chosen to limit the impact of multicollinearity between clade prevalence proportions. The regularisation hyperparameter, alpha, was adjusted independently for each model using the Ridge CV implementation, which uses efficient cross-validation with a single attribute. A wide range of alphas (10^−4^ to 10^4^) was tested in order to select the optimal regularisation strength, thereby minimising prediction error and preventing overfitting. This ensures the generalisation of the reported model coefficients. To enable comparison of the size of the effects, the predictor variables were standardised before model fitting. The standardised regression coefficients can be interpreted as the change in the monthly number of deaths associated with an increase of one standard deviation in the prevalence of the predictor, with all other predictors held constant. The strength of the models was assessed using their R^2^ values.

### 2.3. Demographic Risk Factor Analysis

To identify the demographic groups with the highest or lowest risk of infection linked to each variant, we used a dataset composed of the metadata of 149,148 sequences for which we had the age and sex of the host. Patients were divided into 6 distinct age groups: 0–9, 10–17, 18–29, 30–49 (reference), 50–69, and 70+ years.

As a preliminary step, we performed Pearson’s chi-squared tests to assess the association between variant (defined by clade and lineage) and demographic groups (defined by age group and gender) inside sequenced population. The strength of these associations was quantified using Cramér’s V test, which measures effect sizes for categorical variables.

For each major clade, we fitted a multivariate logistic regression model, using the statsmodels library (v0.14.2) [34], to estimate the Adjusted Odds Ratios (aORs) for each age group (vs. reference Adults 30–49 years) and gender (vs. reference Female). An aOR greater than 1 indicates an increased risk of infection compared with the reference group, taking all factors into account. Significance was determined by 95% confidence intervals that did not include 1.0.

To illustrate the extent of demographic tropism, we carried out a *Z*-score analysis. First, a global reference distribution was established by computing the proportion of all sequenced individuals belonging to each age and gender stratum across the entire dataset. This baseline represents the average demographic profile of the sequenced cohort in Africa. Next, for each clade, we calculated the *Z*-score for each demographic group (defined by gender and age group). A large positive *Z*-score indicates significant over-representation of a clade within the sequenced demographic group.

The *Z*-score was calculated using the standard formula for comparing two proportions, *p*_1_ (the proportion within the clade of interest, on a sample of size *n*_1_) and *p*_2_ (the proportion in the remainder of the dataset, on a sample of size *n*_2_):

$$Z=\left(p_{1}-p_{2}\right)/\surd \left[p_{pool}\times \left(1-p_{pool}\right)\times \left(1/n_{1}+1/n_{2}\right)\right]$$

where *p_pool_* represents the pooled proportion of the demographic group across the entire dataset. A large positive *Z*-score indicates significant over-representation of a clade within the sequenced demographic group, while a negative score indicates under-representation. The results were visualized using a bubble plot.

### 2.4. Phylogenetic Analysis

Phylogenetic reconstruction was performed on 2 subsets using the Nextstrain/Augur bioinformatics pipeline (v28.0.1). One tree was produced using the genomes of all the existing clades, with the number of sequences selected for each clade being proportional to its proportion among the initial dataset. The second tree contains only sequences from the GRA clade associated with Omicron. The genomes were aligned with the Wuhan-Hu-1 reference sequence (NCBI accession number: NC_045512.2) using MAFFT (v7.526), and then a maximum-likelihood phylogeny was produced using IQ-TREE2 (v2.3.6). The resulting trees were time-calibrated using Tree Time (v0.11.4) [34]. The final visualisations were produced using Auspice (v15).

## 3. Results

### 3.1. Diversification of SARS-CoV-2 at the Start of Pandemic

In the beginning of the epidemic in 2020, a total of 17,847 SARS-CoV-2 sequences were recorded across Africa, representing over 100 distinct lineages (Figure S4). While most African countries engaged in genomic surveillance, Namibia, Tchad, and Tanzania did not submit any sequences. The highest sequence contributions came from South Africa, Kenya, Senegal, the Democratic Republic of Congo (DRC), Egypt, and Nigeria, each submitting over 1000 sequences. The B.1 lineage was the most widespread, dominating in 16 countries, including Ethiopia, Kenya, Algeria, DRC, Somalia, Morocco, Cape Verde, Cameroon, Nigeria, and Ghana. It was the first lineage detected in most countries, including Gabon. Meanwhile, the B.1.351 (Beta) lineage was predominantly observed in southern African countries such as Angola, Zimbabwe, South Africa, Mozambique, Malawi, Lesotho, and Eswatini. The B.1.1 lineage was prevalent in Ghana, Gabon, Guinea-Bissau, Nigeria, and Madagascar. Some countries reported only a single lineage due to the limited number of sequences reported. For instance, Sudan reported only lineage A.25, while Liberia submitted just one sequence corresponding to BA.1.15. 

From the onset of the pandemic, most African countries actively engaged in genomic surveillance, which enabled the detection of substantial diversity in variant circulation.

### 3.2. Defining Spatio-Temporal Epidemiological Basins in Africa

**Figure 1:**
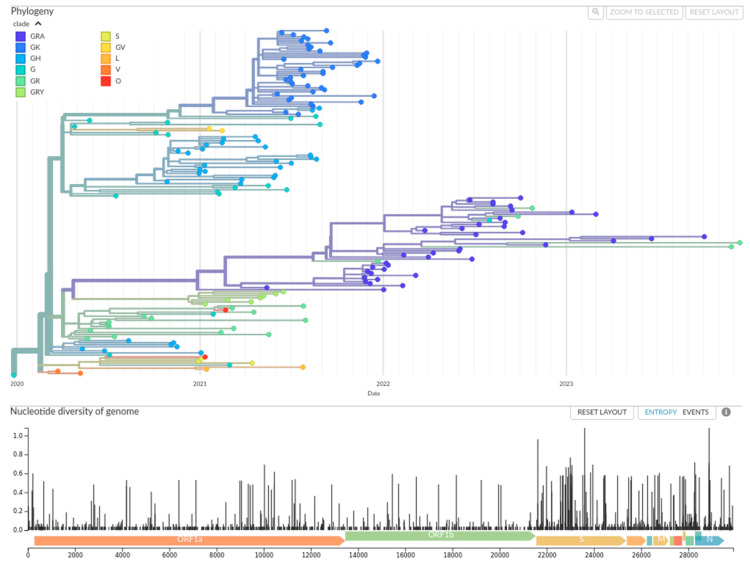
**Successive variant waves defined the SARS-CoV-2 pandemic in Africa**. A time-calibrated maximum likelihood phylogeny of 170 representative SARS-CoV-2 sequences sampled across the African continent between December 2019 and December 2023. The tree is rooted on the Wuhan-Hu-1 reference sequence. The *x*-axis represents time in calendar years, showing the emergence and spread of viral lineages. Tips represent individual sampled genomes, and branches are colored by their assigned Nextstrain clade, as indicated by the legend on the right. The lower panel displays the genomic diversity (Shannon entropy) calculated across the alignment at each nucleotide position, with curated gene annotations (e.g., ORF1a/b, Spike, N) shown above it. The phylogeny clearly illustrates the sequential replacement of early clades by the Beta (GH), Delta (GK), and finally the globally dominant Omicron (GRA) variants, providing a visual timeline for the demographic shifts observed in our risk analysis.

To establish a robust framework for our ecological analyses, we first identified regions sharing a similar pattern of viral circulation during the period of maximum viral diversity (pre-Omicron era, 2020–2021). A Shannon entropy analysis confirms that the diversity of major viral clades was 7.6 times higher during this period compared to the Omicron era that followed (H = 2.46 vs. H = 0.34). A comparative analysis over the entire pandemic also confirmed that including the Omicron era led to less distinct clusters (Figures S1 and S2). This period captures the critical dynamics of the ancestral, Alpha, Beta, and Delta waves, which were subsequently replaced by a more uniform global wave of Omicron (Figure 1).

We performed a Correspondence Analysis (CA) on a contingency matrix of the 30 most frequent Pango lineages among the 102,730 sequences from the pre-Omicron era (2020–2021) in 55 African countries (contingency matrix of lineage distribution detailed in Table S4). This analysis revealed a strong non-random structure in the viral landscape, with the first 5 principal components explaining 62.58% of the total inertia and the first 10 explaining 85.17%. Application of the Elbow method to the coordinates of the first five CA components clearly indicates an optimal partition of k = 7 clusters. K-Means clustering was then used to assign each country to one of these groups (Figure S1) [35].

**Figure 2:**
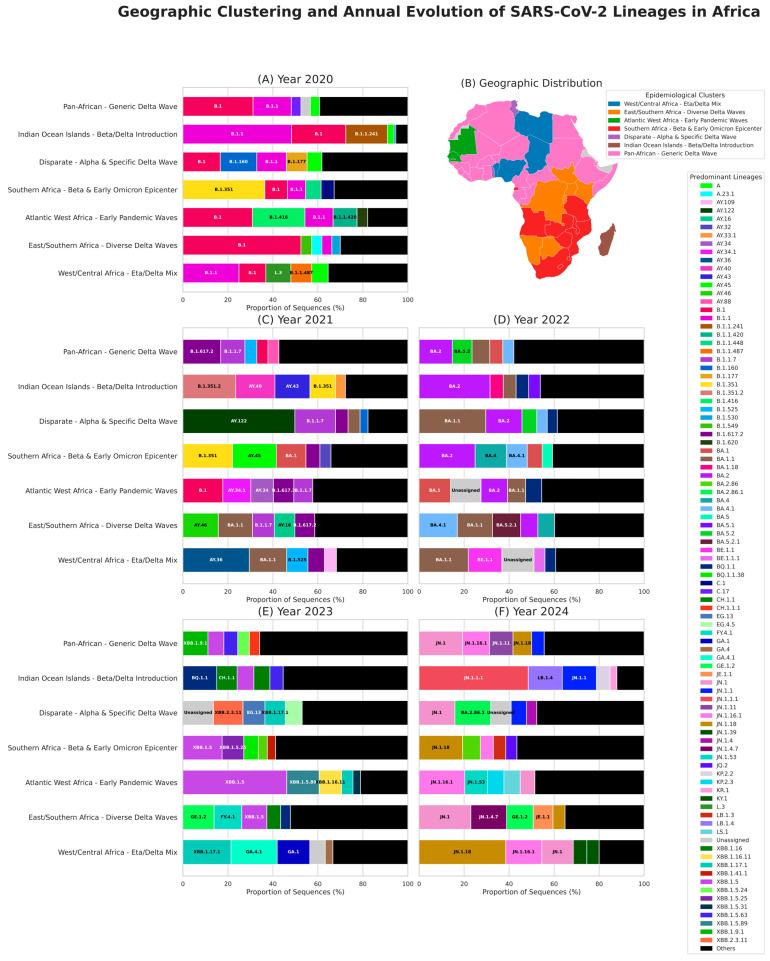
**Lineage-based clustering reveals distinct, temporally evolving epidemiological regions across Africa**. The figure presents a spatiotemporal analysis of SARS-CoV-2 lineage dynamics. (**A**,**C**–**F**) Annual lineage composition for each of the seven epidemiological clusters from 2020 to 2024. Each horizontal bar chart represents a distinct cluster. Segments within each bar show the relative proportion of the top five most frequent lineages identified for that specific cluster and year. All other detected lineages are aggregated into the ‘Others’ category (black). The color of the text overlaying each segment is dynamically chosen for maximal contrast and readability. (**B**) Geographic distribution of the seven epidemiological clusters. These clusters were defined based on viral lineage circulation patterns during the 2020–2021 pre-Omicron period. Each country is colored according to its cluster assignment, providing a visual representation of the distinct epidemiological regions identified through our analysis.

The clusters thus obtained were found to be largely consistent from a geographical and virological point of view. We named them on the basis of their geographical and virological signature: (1) West/Central Africa-Eta/Delta Mix, (2) East/Southern Africa-Diverse Delta Waves, (3) Atlantic West Africa-Early Pandemic Waves, (4) Southern Africa-Beta & Early Omicron Epicenter, (5) Disparate-Alpha & Specific Delta Wave, (6) Indian Ocean Islands-Beta/Delta Introduction, and (7) Pan-African-Generic Delta Wave. Thus, the ‘Southern Africa-Beta & Early Omicron Epicenter’ cluster was defined in this way due to the high dominance of B.1.351 (Beta) and its sub-lineages, and the presence of BA.1 (first Omicron-associated lineage). The ‘Indian Ocean Islands’ cluster only includes nations and regions from the same zone (Comoros, Madagascar, Mayotte, Reunion). The ‘Atlantic West Africa-Early Pandemic waves’ cluster, which includes countries such as Senegal and Gambia, is characterised by the notable presence of the early ancestral lineages B.1 and B.1.1. Pan-Africa cluster, encompassed multiple countries from West, North, and Central Africa. The West/Central Africa cluster includes countries from central North Africa. The East/Southern Africa cluster comprises countries from both southern and eastern Africa. The Disparate cluster includes geographically distant countries from across the continent (Figure 2), clusters characteristics and composition detailed in (clusters virological signatures and member countries are detailed in Table S5 and quantitative clade distributions in Table S6). Additionally, we classified variants into GISAID clades to better capture genomic diversification trends (Figure S5).

Clustering by geographic grouping and clade assignment allowed us to track SARS-CoV-2 evolution across African regions more effectively. This approach also revealed the rapid diversification of the virus within the first year of its emergence, with multiple variants already present in the clades identified in 2020, and enabled monitoring of its progression across successive years of the pandemic. Importantly, this analysis explicitly highlights the five lineages that predominated in Africa, stratified by cluster and year. 

The division of the pandemic into two periods (pre-Omicron and Omicron) enabled us to identify, through correspondence analysis, seven epidemiological basins that shared similar characteristics in variant circulation.

#### 3.2.1. Temporal Dynamics of SARS-CoV-2 in Africa (2020–2024)

##### Continental-Scale Dynamics

At continental level, the pandemic was defined by a series of viral replacement events. Analysis of the prevalence of clades over time shows that the year 2020 was characterised by a competitive mosaic of ancestral clades (G, GH, GR), with no single variant achieving total domination (Figure S5A). This landscape was irrevocably altered in 2021 by the emergence of the GK (Delta-associated) clade, which became dominant in all clusters, representing the first wave of replacement at continental level (Figure S5C). The subsequent emergence of the GRA (Omicron-associated) clade at the end of 2021 marked an even more profound change. In 2022, the GRA clade represented over 83% of the sequences obtained in each of the 7 clusters, even reaching near-total domination (>95%) in regions such as East/Southern Africa (97.3%) and Southern Africa (97.4%). This hegemony persisted through 2023 and 2024 (Figure S5). A time-calibrated phylogenetic tree (Figure 1) visually confirms this history, showing the successive branches of Delta and later Omicron totally eclipsing the earliest and most diverse manifestations of SARS-CoV-2 (Figure S6).

##### Regional Lineage Dynamics

**Figure 3:**
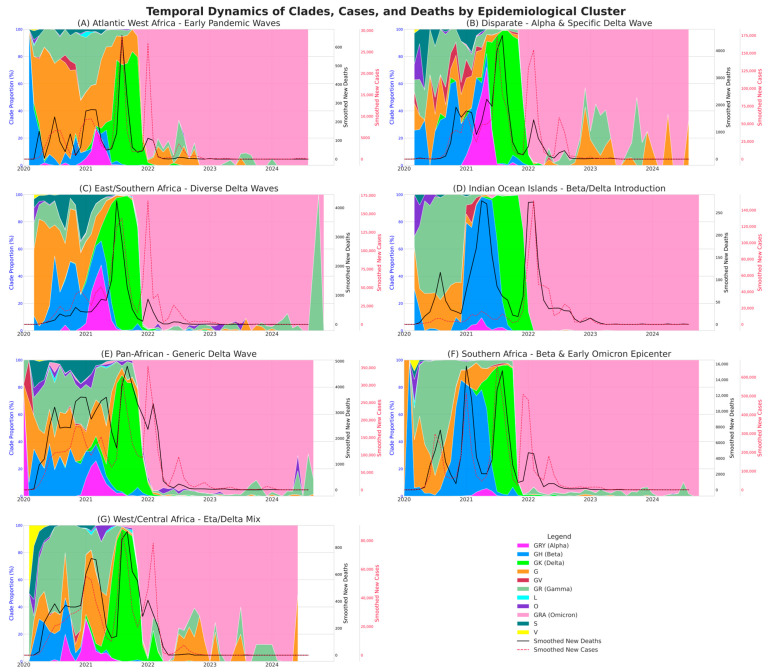
**Coupled dynamics of clade replacement, cases, and deaths reveal regional heterogeneity across African epidemiological clusters**. The figure displays the temporal dynamics of the COVID-19 pandemic across seven distinct epidemiological clusters in Africa, from January 2020 to December 2024. Each panel (**A**–**G**) corresponds to one cluster. Stacked Area Plot (left *y*-axis, blue): The colored areas represent the monthly relative proportion of circulating SARS-CoV-2 clades, derived from sequenced genomes. The color legend maps each color to a specific Nextstrain clade and its corresponding variant name (e.g., GK for Delta). This visualization highlights the successive replacement waves of different viral variants in each region. Line Plots (right *y*-axes): The solid black line shows the 14-day smoothed number of daily new deaths (inner right *y*-axis), while the dashed crimson line represents the 14-day smoothed number of daily new cases (outer right *y*-axis). These lines illustrate the magnitude of the epidemic waves.

While analysis at clade level suggests homogenisation, a more granular view at Pango lineage level reveals a contrasting history of significant and persistent heterogeneity between clusters (Figure 2 and Figure 3). By the start of the pandemic in 2020, clusters were already showing very distinct profiles: “East/Southern Africa” was mainly dominated by the B.1 lineage (52.5%), and “Atlantic West Africa” was dominated by a mix of B.1 (30.9%) and B.1.416 (23.3%). Lineage B.1 and its direct descendant B.1.1 were consistently detected across all clusters, reflecting the early predominance of G and GR clades across Africa. Beta variant and its related lineages established GH clade as the third major clade circulating on the continent during the first year of the pandemic. By the end of 2020, the “Southern Africa” cluster had become an epicentre for the B.1.351 lineage, which reached a 36.4% prevalence in this region (Figure 2A).

In 2021, genomic surveillance expanded significantly, with approximately 84,888 sequences submitted, marking one of the highest levels of participation by African countries. The regionalism of the pandemic became even more pronounced during the Delta wave in 2021. Although monolithic at the clade level, this wave broke down into distinct epidemic regions at a more granular level. Each cluster experienced its own specific Delta wave driven by a different lineage. The “Disparate” cluster (Tunisia, Togo, Seychelles) was almost entirely defined by the AY.122 lineage (49.7% prevalence in 2021). The ‘West/Central Africa’ cluster was characterised by the predominance of AY.36 (29.5%), while the ‘Indian Ocean Islands’ cluster was dominated by a Beta-associated lineage B.1.351.2 (23.4%) alongside multiple Delta-specific lineages such as AY.40 (17.5%) and AY.43 (15.5%). The other clusters all show different predominant lineages, clearly demonstrating the regionalisation of these variants. However, the emergence of Omicron-related lineages with BA.1.1 in East/Southern Africa (15.2%) and West/Central Africa (16.6%) is noteworthy (Figure 2C).

The Omicron era has further amplified this fragmentation. At the beginning of 2022, BA.2 became a major circulating lineage in ‘Southern Africa’ (24.9%), ‘Pan-African’ (14.7%), and ‘Indian Ocean Islands’ (31.5%) while BA.1.1 continued to expand becoming important in ‘West/Central Africa’ (21.9%) and ‘Disparate’ (29.6%) regions. This trend continued into 2023 with XBB.1.5 emerging as dominant ‘Atlantic West Africa’ (46.2%) and ‘Southern Africa’ (17.4%). This landscape of multiple co-circulating lineages persisted through 2023, until a new wave of standardisation occurred in 2024 with the rapid emergence of the JN.1 lineage and its sub-lineages across all regions (Figure 2D,F). This enduring regionalism highlights the fact that our different clusters remained distinct zones of viral evolution during the pandemic (Figure S5).

The 2022–2024 period was characterized by a sharp decline in genomic surveillance, with 53,963 sequences recorded in 2022 and only 14,060 and 2390 in 2023 and 2024, respectively.

##### Temporal Association Analysis

**Figure 4:**
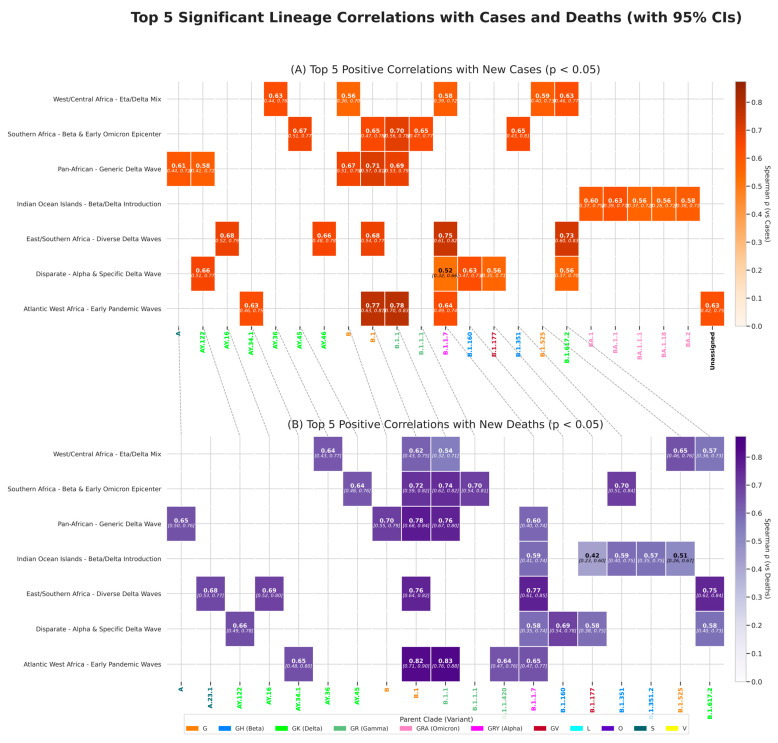
**Heatmap of significant temporal correlations between lineage prevalence and COVID-19 burden**. The figure displays the strongest significant (Spearman’s rank correlation coefficient (ρ), *p* < 0.05) positive correlations between monthly lineage proportions and monthly smoothed case and death counts. (**A**) Correlations with new cases. (**B**) Correlations with new deaths. For each of the seven epidemiological clusters (*y*-axis), the top five lineages demonstrating a significant positive correlation are shown. Cell color and value indicate the Spearman’s rank correlation coefficient (ρ) at the optimal temporal lag (0, 1, or 2 months). Labels for each lineage (*x*-axis) are colored according to their parent Nextstrain clade, providing phylogenetic context. Dashed lines connect common lineages that appear in both panels. The analysis highlights the specific lineages most strongly associated with an increase in COVID-19 burden across different African regions.

To quantify the relationships between the prevalence of variants (clades/lineages) and epidemic waves, we set up a time-lagged Spearman’s rank correlation (ρ) analysis, reinforced by confidence intervals calculated using bootstrap replicates (lineage and clade correlation coefficients with confidence intervals detailed in Tables S7 and S8). The results showed distinct impacts for each major pandemic wave, suggesting a clear hierarchy in their severity (Figure 4 and Figure S7).

The initial waves, led by the ancestral lines, were strongly associated with increased cases and deaths in populations that were still completely immunologically naïve (Figure 3, Figure 4 and Figure S7). One example is the ‘Atlantic West Africa’ cluster, where the rise in lineage B.1.1 (clade GR) showed a very high correlation with deaths (ρ = 0.832, 95% CI [0.755, 0.880]). The G clade, via its B.1 lineage, demonstrated its character as a continental predator, being one of the 5 lineages most correlated with cases in 4 clusters and with deaths in 5 clusters, respectively.

Subsequently, VOCs have become a key element in epidemic dynamics and our analysis shows that certain lineages have acted as threats at the continental level, appearing as drivers of mortality in various epidemiological basins. The Alpha B.1.1.7 lineage is a good illustration of this trend, ranking among the top five lineages most associated with cases in 4 clusters and with deaths in 5 clusters, respectively. Notably, these clusters include ‘East/Southern Africa’ (ρ = 0.768, 95% CI [0.611, 0.849] vs. deaths) and ‘Atlantic West Africa’ (ρ = 0.649, 95% CI [0.472, 0.767] vs. deaths) where the Alpha variant was particularly threatening. The Beta wave (GH clade) also showed a strong association with an increase in the number of deaths, particularly in its epicentre, the ‘Southern Africa’ cluster (lineage B.1.351, ρ = 0.701, 95% CI [0.511, 0.839]). However, even within these generalised continental waves, the devastating Delta wave (GK clade) illustrates a crucial point of our study: it was a composition of distinct regional epidemics. While lineage B.1.617.2 had a fairly global impact (top 5 for cases and deaths in 3 clusters: ‘West/Central Africa’, ‘East/Southern Africa’ and ‘Disparate’), mortality peaks in specific clusters were due to locally successful lineages. For instance, the Delta wave in the “Southern Africa” cluster was primarily driven by the AY.45 lineage (ρ = 0.64, 95% CI [0.48, 0.76] vs. deaths), whereas in “Atlantic West Africa” cluster, it was driven by AY.34.1 (ρ = 0.65, 95% CI [0.48, 0.80] vs. deaths). These results demonstrate the distinct evolutionary paths within our epidemiological basins (Figure 4 and Figure S7).

A definitive change in the narrative occurred with the arrival of the Omicron variant (GRA clade) (Figure 3). This clade was the only one to show a remarkably strong and statistically significant negative correlation with mortality, thus clearly acting as a brake on the pandemic’s lethality (Figure 3, Figure 4 and Figure S7). The signal was most extreme in the ‘Atlantic West Africa’ cluster (ρ = −0.867, 95% CI [−0.915, −0.803] vs. deaths). This observation at a macro level is strongly supported when we look at the dynamics at lineage level. We can cite the example of the ‘Indian Ocean Islands’ cluster, where the 5 lineages most strongly correlated with the increase in the number of cases are all from the clade linked to Omicron, but none of them feature in the top 5 correlations with mortality. This demonstrates the profound change and decoupling between transmission and serious consequences that characterised the Omicron era. 

Finally, the extensive viral diversification observed during the early phase of the pandemic began to slow with the emergence of the Delta variant in 2021. This wave was associated with an increase in both case fatality and overall mortality across the different epidemiological basins, and was characterized by distinct Delta sub-lineages circulating within each basin. It was subsequently followed by the Omicron wave, which, although less lethal, exhibited a substantially higher incidence of cases. 

### 3.3. Main Factors of Severity and Mortality by Region

**Figure 5:**
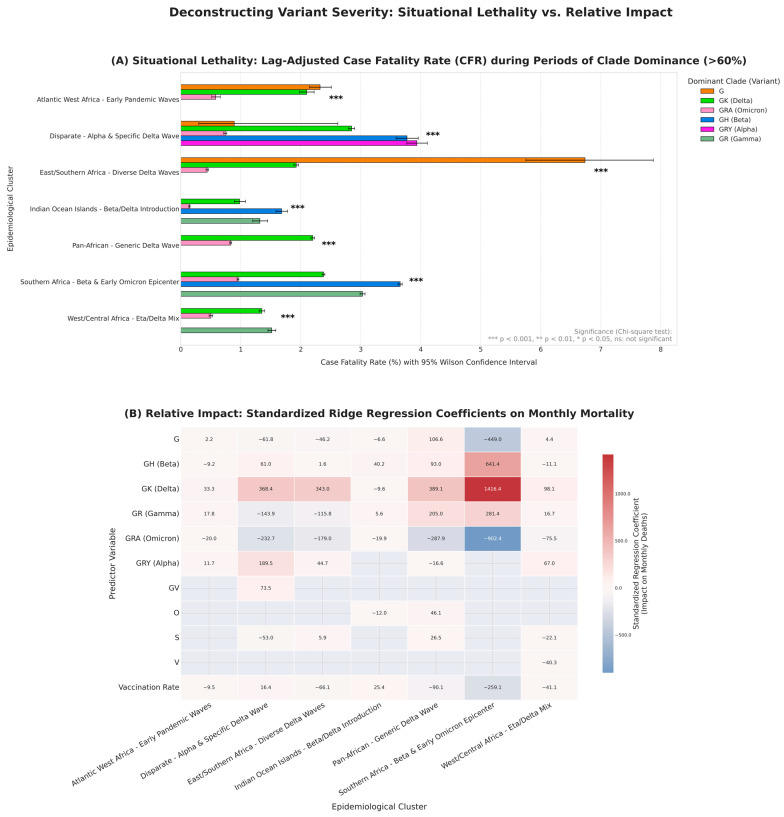
**Deconstructing variant severity: a complementary view of situational lethality and relative epidemiological impact**. (**A**) Lag-Adjusted Case Fatality Rate (CFR) reveals situational lethality. The bar plot shows the CFR calculated for each major clade during months where its prevalence exceeded 60% within each epidemiological basin. The CFR is time-lagged by one month (deaths in month M/cases in month M-1) to account for the typical disease progression timeline. Error bars represent 95% Wilson confidence intervals. Statistical significance of the difference in mortality rates among clades within a single cluster was assessed using a chi-square test (***: *p* < 0.001; **: *p* < 0.01; *: *p* < 0.05; ns: not significant). This panel highlights the high lethality of early clades like G, reflecting the “perfect storm” of a naive population and undeveloped clinical responses. (**B**) Ridge Regression coefficients measure relative impact. The heatmap displays the standardized coefficients from cluster-specific multivariate Ridge regression models, quantifying the impact of each predictor on monthly deaths while controlling for the effects of other co-circulating clades and vaccination rates. Positive coefficients (red) indicate an association with increased mortality, while negative coefficients (blue) suggest a relative protective effect compared to the other variables in the model. Grey cells indicate that a clade was excluded from the model for that specific cluster due to its low prevalence, ensuring model robustness. This panel correctly identifies Delta (GK) as the primary driver of mortality and contextualizes the impact of other variants relative to it.

To disentangle the impact of each variant while taking into account co-circulating clades and the effect of vaccination, we fitted a robust multivariate Ridge regression model to each cluster (Figure 5B, full regression model coefficients and statistics detailed in Table S9). The results clearly identify the Delta-associated clade (GK) as the main driver of mortality across the continent during the pandemic, confirming our previous findings. Its prevalence was strongly associated with an overall increase in the number of deaths. The largest standardised coefficients were found in the ‘Pan-African’ (+389.1), “Disparate” (368.4) and ‘East/Southern Africa’ (343.0) clusters. In contrast, the Omicron-related clade (GRA) was consistently associated with a significant relative reduction in mortality, particularly in the ‘Southern Africa’ cluster (coefficient: −902.4). Some clades had more heterogeneous impacts on the increase in the number of deaths depending on the cluster. Clade G had impacts ranging from very negative (‘Southern Africa’ (coefficient: −449.0) to little effect, with only one real positive correlation in the ‘Pan-African’ cluster (+106.6). Clade GH (Beta) had a particularly positive impact (i.e., a strong association with an increase in deaths) in the Indian Ocean Islands (+40.2) and Southern Africa (+641.4) clusters. The GR (Gamma) clade had a divergent, region-dependent effect. It was one of the two clades most strongly associated with increased mortality in ‘Atlantic West Africa’ and ‘Pan-African’ clusters. Yet, in the “Disparate” and ‘East/Southern Africa’ clusters, its association with deaths was minimal.

The model also suggests a robust overall effect of vaccination, with vaccination rates showing a strong negative association with mortality, particularly in the ‘Southern Africa’ (coefficient: −259.1) and ‘Pan-African’ (−90.1) clusters. However, the magnitude of the negative regression coefficients for GRA (Omicron) was systematically larger than that for vaccination. This suggests that the replacement of other variants by Omicron as well as other factors not considered in this model may have had a greater impact on the decline in mortality than the increased vaccination rates in these populations. It is also worth noting that the ‘Southern Africa’ cluster is unusual in terms of the wide range of its values, and the model reveals the exceptional severity of its major waves. The impact of the Delta variant (GK clade) in this cluster (+1416.4) was by far the greatest observed across all regions, reinforcing the status of this region as the epicentre of extreme pandemic dynamics. Overall, the models performed well for ecological data, with R^2^ values ranging from 0.44 to 0.68.

While the regression model identifies the impact of each variant on the pandemic as a whole, our robust, time-lagged Case Fatality Rate (CFR) analysis provides a complementary measure of lethality (Figure 5A, sensitivity analysis of CFR across varying dominance thresholds detailed in Table S10). This analysis reveals a somewhat different hierarchy of severity. This analysis highlights the fact that the highest CFR was observed not during the Delta wave but during the ancestral G clade wave within the “East/Southern Africa” cluster, reaching an exceptional value of 6.74% (95% CI: 5.75–7.88). These results do not necessarily reflect the intrinsic virulence of the variant, but rather its devastating impact in a particular initial pandemic context. Infections occurred in an immunologically naive population, while testing capacities were still limited, biasing the case count and thus the calculation of the CFR.

Thus, the two analyses are not contradictory but complementary. The high CFR for clade G quantifies lethality in an early pandemic scenario, while the negative value of the coefficient for this same clade measures its less pronounced impact on mortality when compared to the catastrophic Delta wave. Overall, the Alpha variant (GRY) waves show the highest average CFR (3.94%), followed by the ancestral clades G (3.32%) and GH (Beta-related, 3.04%). All of these appear to be more severe than the Delta wave (GK, 1.97%) based on these raw CFR values. In stark contrast, while consistent with the regression models, the lowest CFR was recorded during periods of Omicron (GRA) dominance in the “Indian Ocean Islands” cluster (0.14%; 95% CI: 0.13–0.16), a CFR more than 48 times lower than that of the peak of the G clade. Finally, chi-square tests confirm that the observed differences within clusters are not random artefacts, as they are all highly significant within each cluster (*p* < 0.001). 

Together, these two analyses corroborate our previous findings regarding the deadly Delta wave and suggest a potential protective effect of vaccination, particularly during the Omicron wave.

### 3.4. Variant-Specific Demographic Risk Profiles

**Figure 6:**
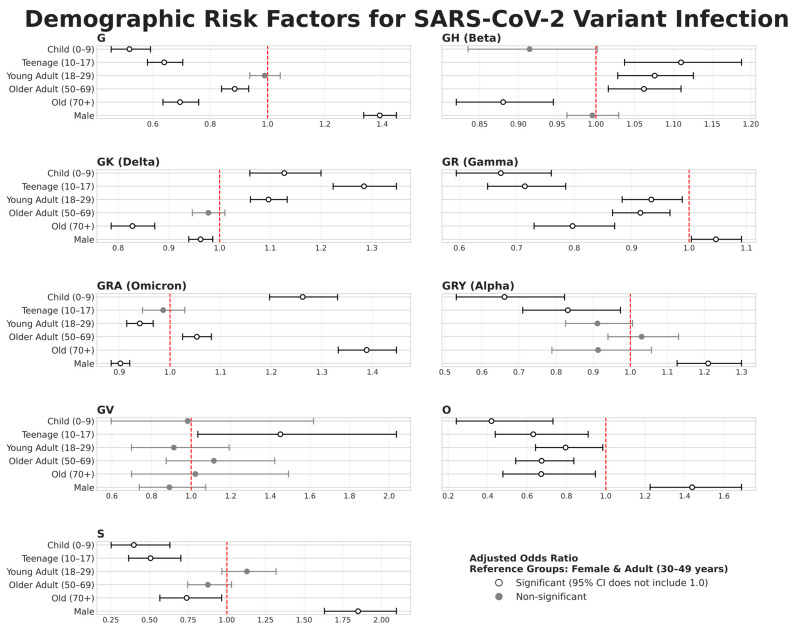
**Demographic Risk Factors for Infection by SARS-CoV-2 Variant**. Forest plot displaying the Adjusted Odds Ratios (aORs) for demographic risk factors associated with specific SARS-CoV-2 clades. For each clade, a separate multivariate logistic regression model was fitted, controlling for both age and gender. The outcome variable was the probability of a sequence belonging to the indicated clade. Circles represent the point estimate of the aOR, and horizontal lines indicate the 95% confidence intervals. The vertical dashed red line at OR = 1.0 signifies no effect. An aOR is statistically significant if its confidence interval does not cross this line. The reference groups for comparison are Females and the Adult (30–49 years) age category. Only clades with a minimum of 200 observations were included in the analysis.

In order to characterise the impact of the pandemic among different demographic groups, we analysed demographic data from 149,148 sequences. Although an initial Pearson’s chi-squared test showed a highly significant association between clade and host demographics (*p* < 10^−139^), we note that the effect size was small (Cramér’s V < 0.1). This suggests that these global metrics may mask stronger evolutionary dynamics at the variant level. By performing both multivariate logistic regression (Figure 6, multivariate logistic regression outputs and adjusted odds ratios detailed in Table S11) and *Z*-score analysis (Figure S7, complete demographic *Z*-score analysis detailed in Table S12), we revealed three distinct patterns of viral transmission and demonstrated a dramatic and evolving change in the profile of at-risk populations.

#### 3.4.1. The Ancestral and Alpha Wave

The initial phase of the pandemic, dominated by ancestral clades (G, S) and the Alpha (GRY) variant, established a specific risk profile in which young men were overrepresented. This profile was most pronounced for the G clade, for which the odds of a sequenced case being male were 39% higher than for a female case (Adjusted Odds Ratio: 1.39, 95% CI [1.34–1.45]) (Figure 6). Our *Z*-score analysis allowed us to better quantify the magnitude of these differences: the over-representation of men aged 30–49 within clade G reached a *Z*-score of +15.33 (Figure S8). This is a value of exceptional statistical significance, representing the most intense and unambiguous demographic signal observed across our entire dataset. In contrast, this wave was marked by a significant protective effect for young people, with children (aOR: 0.52, 95% CI [0.45–0.59]) and teenagers (aOR: 0.64, 95% CI [0.58–0.70]) being significantly under-represented.

#### 3.4.2. The Delta Wave

The arrival of the Delta-related clade GK has not only changed this risk profile; it has fundamentally reversed it. The burden of infection has shifted towards younger populations. Teenagers (10–17 years) have become the group most at risk, with a 28% higher chance of infection than adults (aOR: 1.28, 95% CI [1.22–1.35]), closely followed by children (0–9 years; aOR: 1.13, 95% CI [1.06–1.20]). The *Z*-score analysis identified the new epicentre of transmission: the strongest positive signal for Delta was now women aged 10–17 (*Z*-score: +8.91). For the first time in the pandemic, the risk associated with men disappeared, indicating a modest yet significant protective effect (aOR: 0.96, 95% CI [0.94–0.99]).

#### 3.4.3. The Omicron Wave

The Omicron wave (GRA) established a new paradigm characterised by its U-shape or bimodal age profile. The risk became more strongly concentrated at the extremes of the age spectrum, the oldest group (70+ years) experienced the most dramatic increase in their risk (aOR: 1.39, 95% CI [1.33–1.45]), while a new risk emerged for young children (0–9 years; aOR: 1.26, 95% CI [1.20–1.33]). Groups most affected by previous waves were now relatively protected. This reduction in risk in middle-aged populations was clearly illustrated by the *Z*-score for men aged 18–29, which fell to −12.13, the most intense underrepresentation of any group during the pandemic. This demonstrates a significant shift in the affected populations, driven by viral evolution and population immunity.

Although these results demonstrate changes caused by circulating variants, it is important to note that these profiles are based on the populations for which we had sequencing data. They may also be influenced by other factors such as vaccination trends within populations, acquired immunity, and non-pharmaceutical interventions within populations.

## 4. Discussion

Our continent-wide genomic analysis challenges the notion of a uniform pandemic in Africa. Rather than passively undergoing global viral waves, our data reveal the existence of distinct epidemiological basins across the continent, each characterized by unique viral signatures and severity dynamics. Table S13 provides a summary of the characteristics identified in our study of the different circulating clades/variants. We identified three key conclusions: (i) viral evolution in Africa is highly regionalized; (ii) the “severity” of a variant is defined by both geographical context and relative impact; and (iii) the demographic risk landscape has been profoundly reshaped by the interplay between viral evolution and population immunity.

The identification of seven stable epidemiological basins, each with a specific lineage signature, undermines the notion of a single pandemic wave in Africa. Between June 2020 and August 2021, a period during which several African countries experienced their major COVID-19 waves [36] continental sequencing data revealed a virus diversified into multiple clades, with numerous descendants and country-specific predominance of certain variants, as highlighted by genomic surveillance studies across Africa [36,37,38].

The pre-Omicron period showed that transmission was occurred through geographical and logistical corridors rather than border proximity. The Delta wave is a striking example. While appearing as a global single event linked to the GK clade, it was in fact a mosaic of lineage-specific epidemics, with AY.45 dominating in Southern Africa, whereas AY.34.1 predominated in West Africa. This shifts the narrative from a simple importation model toward one of regional emergence and selection [11,19,39].

Thus, cluster comparisons describe a quasi-random grouping of countries with similar epidemiological trajectories [13,16]. This dynamic may partly be explained by Africa’s high human genetic diversity, the greatest worldwide [11,40].

Our analysis addresses the crucial question of how to measure the “severity” of a variant. We clarify the apparent paradox of the ancestral G clade, which exhibited the highest case fatality rate (CFR) in our dataset reaching 6.74% in one cluster yet recorded a negative coefficient in our regression model. These findings are not contradictory; they capture distinct phenomena.

The elevated CFR of clade G reflects situational lethality, i.e., its strong impact at the beginning of 2020 on an immunologically naïve population, in a context where testing primarily targeted severe cases and no effective treatments were available [3,12]. Conversely, the negative regression coefficient quantifies its relative impact on mortality once the effects of all other variants are considered. The model identifies Delta as the main driver of mortality on the continent throughout the period [36,41].

The virus host interaction has been dynamic, with notable trends in the impact of different SARS-CoV-2 clades across age and sex groups in Africa, and distinct patterns over the course of the pandemic. We identified three successive demographic regimes. Certain clades disproportionately affected specific demographic segments, suggesting possible reinfections and highlighting the virus’s evolving nature [1,19,42,43].

Early pandemic waves (ancestral and Alpha) followed a classical risk profile, concentrating infections among adult men (aOR: 1.39), likely due to occupational exposure and mobility [18,44]. The emergence of Delta marked a fundamental shift, with the burden of infection moving toward younger populations and women. Teenage girls (10–17 years) thus became a high-risk group [19].

As infections and reinfections progressed, immunity was acquired within the population. However, the virus continued to adapt to its host, so synergistic interactions between mutations became increasingly important [45,46,47]. The Omicron wave established a third regime with a U-shaped risk profile, disproportionately affecting both young children (0–9 years) and the elderly (≥70 years). This trajectory suggests continuous viral adaptation, exploiting demographic niches least protected by immunity from earlier waves, leading to the inclusion of pediatric vaccination, particularly for children aged 5–11 [12,48].

Despite reflecting global trends [49,50], Africa reported relatively fewer COVID-19 deaths. In 2021, Africa accounted for less than 4% of global deaths [16]. Despite weaker health systems, the continent had the world’s lowest mortality rate [42], with limited vaccine coverage [24]. In Western countries such as the United States and France, individuals over 70 years bore the highest mortality, while in Gabon it was those over 65, and in Congo, individuals aged 30–49 [51]. Africa’s young demographic profile may partly explain these variations [52]. Additional contributing factors include biodiversity, zoonotic exposure through frequent contact with wildlife [1,9], pre-existing cross-reactive humoral immunity to SARS-CoV-2 documented in Gabon five years prior to the pandemic [53], widespread self-medication practices biasing case and death reporting [54], and environmental factors such as humidity and high temperatures limiting viral stability [44,46].

Omicron (B.1.1.529) and its numerous sublineages (BA.1, BA.2, BA.5, XBB, BQ.1, CH.1.1, EG.5, JN.1) emerged between 2021 and 2024, illustrating the ongoing evolutionary dynamics of SARS-CoV-2 [55,56]. Our phylogenetic analysis produced a provocative finding: the presence of sequences classified as GRA (Omicron) in Mauritius as early as June 2020 well before its official detection in Southern Africa in late 2021 [57]. If confirmed and not due to metadata errors, this raises the fascinating possibility of a long cryptic circulation and evolution of Omicron on the continent, undetected by the scientific community [6]. More broadly, this underscores the limitations of global genomic surveillance and highlights the urgent need for massive, sustainable investment in equitable regional surveillance networks [3,42].

Furthermore, unlike other clades, the emergence and predominance of Omicron sublineages toward the end of the pandemic was accompanied by reduced lethality despite increased transmissibility, a phenomenon also observed in many other regions [45,58].

### 4.1. Study Limitations

Our findings must be interpreted within the methodological framework of the study and the complexities inherent in continental-scale pandemic data. Defining epidemiological basins based on pre-Omicron data was effective for capturing the foundational dynamics of the pandemic, but transmission corridors may have evolved during the Omicron era [24,59]. Likewise, our demographic risk profiles are based on sequenced populations, which do not necessarily reflect all infected individuals, and the absence of individual-level clinical data prevents direct linkage to severity [3,12]. Broader epidemiological metrics are also subject to real-world biases. The high CFR observed for early variants was likely influenced by limited testing capacity early in the pandemic [3], which biased detection toward severe and hospitalized cases. These local biases are amplified by marked heterogeneity in genomic surveillance across Africa, where despite commendable efforts, entire regions remain genomic “blind spots,” potentially obscuring variant emergence and early evolution. At the same time, the significant contributions of a few nations such as South Africa and Nigeria carry disproportionate weight in analyses [19,37,60]. Conversely, the well-documented decline in sequencing efforts during the Omicron wave may have affected prevalence estimates for this period [9,13].

Although our models provide a robust statistical framework, their scope remains defined by available variables. Factors such as non-pharmaceutical interventions, population behaviors, and comorbidity prevalence were not included, yet they undoubtedly shaped local mortality and transmission patterns [52]. Moreover, a strong correlation between the rapid expansion of a lineage and increased deaths does not rule out the influence of competing variants [38]. Our study clearly provides data regarding SARS-CoV-2 lineages and their localisation in Africa. However, additional data such as mobility and human interactions would allow to elucidate several aspects of the virus transmission and the evolution of the pandemic in Africa.

### 4.2. COVID-19 Pandemic Management

The initial spread of SARS-CoV-2 in Africa was likely driven by imported cases, particularly from Europe, followed by regional transmission [12]. Support from international agencies such as the Africa CDC and WHO through the provision of sequencing equipment, training, and vaccine donations was critical in managing the crisis [3,12].

Genomic surveillance played a pivotal role in tracking viral evolution and managing the COVID-19 pandemic [14,42], particularly in the face of increasing reinfections driven by viral mutations [50].

Vaccines demonstrated efficacy against the first variants of concern from 2021 onward. However, breakthrough infections occurred among vaccinated individuals [43]. Vaccine hesitancy contributed to resurgences, particularly among the unvaccinated and immunocompromised [59]. Omicron emerged with more than 50 mutations, likely influenced by immune pressure from global vaccination campaigns [59]. While vaccination may have contributed to Omicron’s emergence, booster doses significantly curtailed its spread, with mRNA vaccines achieving around 90% efficacy even against the most recent variants [61]. Nonetheless, vaccine uptake in Africa remained low, as in Gabon where only 13% of the population accepted vaccination, mainly due to hesitancy [24,59].

## 5. Conclusions

This study outlines the evolutionary trajectory of SARS-CoV-2 in Africa, showing early viral diversification followed by the emergence of highly transmissible and immune-evasive variants. Our findings revealed that variant circulation during epidemic waves was not homogeneous across the continent and highlighted the intra-continental dynamics by the appearance of regional clusters and the description of epidemiological basins. Future efforts should prioritize expanding genomic surveillance, improving data-sharing, and studying virus–host dynamics to better prepare for future epidemics and emerging viral threats in Africa.

## Data Availability

Data is contained within the article or Supplementary Material.

## Supplementary Materials

The following supporting information can be downloaded at: https://www.mdpi.com/article/10.3390/v18050524/s1. Supplementary Methods S1, Detailed Worked Examples for Demographic Analyses. Table S1, Geotemporal distribution of high-quality SARS-CoV-2 genomic sequences; Table S2, Sensitivity analysis of clustering stability across optimal parameters; Table S3, Annual and Cumulative SARS-CoV-2 Cases and Deaths for the Africa Study Cohort; Table S4, Pre-Omicron lineage-country contingency matrix; Table S5, Characteristics and virological signatures of the seven epidemiological basins; Table S6, Distribution of major SARS-CoV-2 clades across the seven epidemiological basins; Table S7, Significant Spearman correlations between lineage prevalence and epidemiological indicators; Table S8, Summary of Spearman correlations between clade prevalence and epidemiological indicators; Table S9, Complete results of the cluster-specific Ridge Regression models; Table S10, Sensitivity analysis of the time-lagged Case Fatality Rate (CFR); Table S11, Complete results of the multivariate logistic regression analysis of demographic risk factors; Table S12, Comprehensive *Z*-score analysis of demographic tropism by clade; Table S13, Comprehensive summary of the major Nextstrain clades observed in Africa during 2020–2024 period. Figure S1, Defining lineage-based epidemiological clusters in Africa using Correspondence Analysis; Figure S2, Comparative clustering analysis on the full pandemic period reveals the homogenizing effect of Omicron; Figure S3, Systematic validation of clustering parameters; Figure S4, The dominance of B.1 at continental level and the diversity of circulating lineages in early stages of SARS-CoV-2 pandemic; Figure S5, Clusters clade evolving distribution in our lineage-based clustering study; Figure S6, Omicron (GRA clade) phylogenetic tree; Figure S7, Optimal time-lagged correlations between SARS-CoV-2 clade prevalence and epidemiological impact across African clusters; Figure S8, Variant-specific demographic profiles reveal a shift in age tropism during the SARS-CoV-2 pandemic in Africa.
