# Supplementary material for: Dual Aspect of the Pandemic on the African Continent: Viral Distribution and Shifting Demographic Susceptibility to SARS-CoV-2
Source: Viruses. 2026 Apr 30;18(5):524. doi: 10.3390/v18050524 (PMC13211388; doi:10.3390/v18050524)
Supplement: Supplementary file 1 [file viruses-18-00524-s001.zip › viruses-3981074-supplementary 10 12 25/viruses-3981074-supplementary files/viruses-3981074-supplementary.docx]

**Dual Aspect of the Pandemic on the African Continent: Viral
Distribution and Shifting Demographic Susceptibility to
SARS-CoV-2**

**Julia Cyrielle Andeko ^1,†^, Sonia Etenna Lekana-Douki ^1,^*^,†^, Gabriel Falque ^1^, Nadine N’dilimabaka ^1,2,3^
and Jean-Bernard Lekana-Douki ^1,4^**

^1^ Centre Interdisciplinaire de Recherches Médicales de Franceville (CIRMF), Franceville BP 769, Gabon;
juliatesse@gmail.com (J.C.A.); gabfalque@hotmail.fr (G.F.); nadinendilimabaka@yahoo.fr (N.N.);
lekana_jb@yahoo.fr (J.-B.L.-D.)

^2^ Département de Biologie, Faculté des Sciences, Université des Sciences et Techniques de Masuku (USTM), Franceville BP 901, Gabon

^3^ Ecole des Sciences et Medecine Vétérinaire de Masuku (ESMVM), Université des Sciences et Techniques de Masuku (USTM), Franceville BP 901, Gabon

^4^ Université des Sciences de la Santé, Libreville BP 4009, Gabon

* Correspondence: s_lekana@yahoo.fr

^†^ These authors contributed equally to this work.

# Supplementary Files

## Supplementary Methods:

### Supplementary Methods S1: Detailed Worked Examples for Demographic Analyses

This section provides a transparent, step-by-step illustration of the key statistical methods used for the demographic risk factor analysis presented in sections 2.3 and 3.4 of the main text. We provide a worked example for both the Adjusted Odds Ratio (aOR) and the demographic Z-score calculations, using actual data points from our study to ensure clarity and reproducibility.

#### S1.1 Adjusted Odds Ratio (aOR) from Multivariate Logistic Regression

**Objective**

The goal of this analysis is to isolate and quantify the association between a specific demographic characteristic (e.g., being male) and the probability of infection with a particular viral clade, while statistically controlling for the confounding effects of other variables (e.g., age). The result, an Adjusted Odds Ratio (aOR), provides a measure of relative risk.

**Methodological Framework**

For each major clade, we fitted a multivariate logistic regression model. The model's outcome variable is binary: 1 if a sequence belongs to the clade of interest, and 0 otherwise. The predictors are dummy-coded variables representing demographic strata (six age groups and two genders). To avoid perfect multicollinearity, one category from each variable is held out as a reference: 'Adult (30-49 years)' for age and 'Female' for gender.

The model estimates a set of coefficients (β), known as log-odds, for each predictor. These coefficients represent the change in the log-odds of the outcome for a one-unit change in the predictor, holding all other predictors constant.

**Worked Example: Quantifying the Increased Infection Risk for Males in the Ancestral G Clade**

This example demonstrates how the aOR of 1.39 for the sex_Male variable in the G clade model was calculated (as seen in Figure 6 and Supplementary Table S9).

*Model Specification & Context:*

Outcome: A sequence belonging to clade G (1) vs. any other clade (0).

Predictors: age_child (0-9), age_teenage (10-17), ..., sex_Male.

Reference Groups: age_adult (30-49) and sex_Female.

*Coefficient (β) Extraction:*

After fitting the model, we extract the raw coefficient (the log-odds) for our variable of interest, sex_Male. This value represents the model's estimate of the effect of being male, adjusted for age.

Coefficient (β) for sex_Male: 0.3299

*Calculating the Adjusted Odds Ratio (aOR):*

The aOR is the exponentiation of the β coefficient. This transforms the log-odds into a more intuitive ratio of odds.

aOR = e^β = e^0.3299 ≈ 1.3908

*Deriving the 95% Confidence Interval (CI):*

The model also provides a 95% CI for the β coefficient. By exponentiating these bounds, we obtain the CI for the aOR, which quantifies the uncertainty around our estimate.

95% CI for β: [0.2890, 0.3708]

95% CI for aOR = [ e^0.2890, e^0.3708 ] = [1.3351, 1.4489]

**Interpretation Guide for Figure 6 (Forest Plot)**

This calculated result is visualized directly in the forest plot for the 'G' clade.

The circle on the 'Male' row is plotted at the aOR value of 1.39.

The horizontal line (error bar) extending from the circle represents the 95% CI, spanning from 1.34 to 1.45.

Because this entire interval is to the right of the vertical dashed line at 1.0, we conclude the result is statistically significant, indicating that males had a significantly higher odds of infection with the G clade compared to females, even after accounting for age.

#### S1.2 Demographic Z-score for Proportional Representation

**Objective**

The goal of this analysis is to determine if a demographic group is significantly over- or under-represented within a specific viral clade compared to its baseline proportion across all other clades combined. Unlike the aOR, this is a direct comparison of proportions and does not adjust for other variables.

**Methodological Framework**

We used a Z-score test for two independent population proportions. This test evaluates the null hypothesis that the proportion of a demographic group in a clade (p₁) is the same as its proportion in the rest of the dataset (p₂). The resulting Z-score measures how many standard errors the observed difference (p₁ - p₂) is from zero. An absolute Z-score greater than 1.96 (for α=0.05) is considered statistically significant.

**Worked Example: The Exceptional Over-representation of Males (30-49 years) in the G clade**

This example details the calculation of the Z-score of +15.33, the most intense demographic signal detected in our study.

*Data Definition & Counts:*

Population 1 (Clade G):

Total sequences in Clade G (n₁): 9,983

Number of males aged 30-49 in Clade G (x₁): 2,484

Population 2 (All Other Clades):

Total sequences in the rest of the dataset (n₂): 139,165

Number of males aged 30-49 in the rest of the dataset (x₂): 25,945

*Proportion Calculation:*

Proportion in Clade G (p₁) = x₁ / n₁ = 2,484 / 9,983 ≈ 0.2488

Proportion in Other Clades (p₂) = x₂ / n₂ = 25,945 / 139,165 ≈ 0.1864

*Standard Error Calculation:*

First, we calculate the pooled proportion (p_pool), which is the overall proportion of the group in the entire dataset.

p_pool = (x₁ + x₂) / (n₁ + n₂) = (2,484 + 25,945) / (9,983 + 139,165) ≈ 0.1906

The Standard Error (SE) of the difference is then: SE = √[ p_pool * (1 - p_pool) * (1/n₁ + 1/n₂) ]

SE = √[ 0.1906 * (0.8094) * (1/9983 + 1/139165) ] ≈ 0.00406

*Final Z-score Calculation:*

Z = (p₁ - p₂) / SE

Z = (0.2488 - 0.1864) / 0.00406 ≈ +15.33

**Interpretation Guide for the Bubble Plot**

This Z-score is visualized in the bubble plot corresponding to the intersection of the 'G' clade and the '30-49' age group for males.

The color of the bubble is red (or the positive color) because the Z-score is > 0.

The size of the bubble is directly proportional to the absolute magnitude of the Z-score. A value of 15.33 results in a very large bubble, visually representing the exceptional strength of this demographic signal.

## Supplementary Tables :

### Table S1: Geotemporal distribution of high-quality SARS-CoV-2 genomic sequences.

Table S1: **Geotemporal distribution of high-quality SARS-CoV-2 genomic sequences.** The table provides a summary of the number of high-quality SARS-CoV-2 genomes analyzed in this study, aggregated by country of origin (rows) and year of collection (columns). Counts were derived from the final curated metadata dataset, encompassing a total of 173148 genomes from 55 African nations. The ‘Total country’ column provides the cumulative count of sequences for each nation across the entire study period. The ‘Total year’ rows, displayed at the top and bottom for reference, provide the continent-wide sum of sequences for each respective year, outlining the temporal sampling intensity of the genomic surveillance effort.

|  | **2020** | **2021** | **2022** | **2023** | **2024** | **Total country** |
| --- | --- | --- | --- | --- | --- | --- |
| **Total year** | 17847 | 84888 | 53963 | 14060 | 2390 | 173148 |
| **Algeria** | 94 | 229 | 555 | 195 | 142 | 1215 |
| **Angola** | 151 | 1095 | 89 | 0 | 0 | 1335 |
| **Benin** | 12 | 1126 | 167 | 1 | 0 | 1306 |
| **Botswana** | 80 | 2922 | 2287 | 82 | 19 | 5390 |
| **Burkina Faso** | 395 | 385 | 81 | 5 | 0 | 866 |
| **Burundi** | 0 | 157 | 0 | 0 | 0 | 157 |
| **Cabo Verde** | 13 | 335 | 479 | 216 | 4 | 1047 |
| **Cameroon** | 118 | 921 | 737 | 13 | 21 | 1810 |
| **Central African Republic** | 10 | 121 | 71 | 13 | 6 | 221 |
| **Chad** | 0 | 68 | 23 | 0 | 0 | 91 |
| **Comoros** | 0 | 34 | 8 | 0 | 0 | 42 |
| **Cote d’Ivoire** | 116 | 664 | 167 | 87 | 53 | 1087 |
| **Democratic Republic of the Congo** | 539 | 691 | 183 | 22 | 23 | 1458 |
| **Djibouti** | 59 | 432 | 515 | 8 | 0 | 1014 |
| **Egypt** | 807 | 1130 | 2447 | 547 | 54 | 4985 |
| **Equatorial Guinea** | 139 | 73 | 1 | 0 | 0 | 213 |
| **Eswatini** | 17 | 753 | 444 | 95 | 0 | 1309 |
| **Ethiopia** | 8 | 555 | 255 | 1 | 0 | 819 |
| **Gabon** | 184 | 792 | 52 | 0 | 0 | 1028 |
| **Gambia** | 418 | 743 | 253 | 0 | 0 | 1414 |
| **Ghana** | 305 | 3377 | 1577 | 90 | 122 | 5471 |
| **Guinea** | 182 | 383 | 300 | 68 | 1 | 934 |
| **Guinea-Bissau** | 8 | 40 | 0 | 0 | 0 | 48 |
| **Kenya** | 2324 | 7256 | 3154 | 625 | 182 | 13541 |
| **Lesotho** | 15 | 174 | 91 | 21 | 0 | 301 |
| **Liberia** | 3 | 153 | 16 | 0 | 0 | 172 |
| **Libya** | 24 | 125 | 32 | 0 | 0 | 181 |
| **Madagascar** | 369 | 458 | 53 | 50 | 31 | 961 |
| **Malawi** | 68 | 1190 | 189 | 21 | 0 | 1468 |
| **Mali** | 59 | 187 | 147 | 15 | 0 | 408 |
| **Mauritania** | 4 | 118 | 20 | 0 | 0 | 142 |
| **Mauritius** | 46 | 843 | 5674 | 2362 | 293 | 9218 |
| **Mayotte** | 0 | 926 | 304 | 10 | 1 | 1241 |
| **Morocco** | 309 | 543 | 1506 | 178 | 7 | 2543 |
| **Mozambique** | 165 | 1085 | 426 | 308 | 14 | 1998 |
| **Namibia** | 0 | 582 | 225 | 55 | 2 | 864 |
| **Niger** | 104 | 125 | 158 | 21 | 0 | 408 |
| **Nigeria** | 458 | 6025 | 1375 | 116 | 330 | 8304 |
| **Republic of the Congo** | 71 | 513 | 125 | 19 | 0 | 728 |
| **Reunion** | 4 | 9600 | 9436 | 2643 | 1 | 21684 |
| **Rwanda** | 184 | 548 | 55 | 0 | 0 | 787 |
| **Sao Tome and Principe** | 0 | 10 | 0 | 0 | 0 | 10 |
| **Senegal** | 1090 | 3486 | 1629 | 83 | 134 | 6422 |
| **Seychelles** | 4 | 1058 | 490 | 0 | 0 | 1552 |
| **Sierra Leone** | 18 | 110 | 0 | 0 | 1 | 129 |
| **Somalia** | 24 | 18 | 3 | 10 | 0 | 55 |
| **South Africa** | 7345 | 27690 | 16069 | 5244 | 830 | 57178 |
| **South Sudan** | 1 | 169 | 0 | 0 | 11 | 181 |
| **Sudan** | 28 | 306 | 222 | 6 | 0 | 562 |
| **Tanzania** | 0 | 31 | 0 | 0 | 0 | 31 |
| **Togo** | 47 | 604 | 432 | 32 | 0 | 1115 |
| **Tunisia** | 208 | 1731 | 513 | 234 | 44 | 2730 |
| **Uganda** | 399 | 762 | 593 | 374 | 59 | 2187 |
| **Zambia** | 489 | 837 | 255 | 172 | 5 | 1758 |
| **Zimbabwe** | 332 | 599 | 80 | 18 | 0 | 1029 |
| **Total year** | 17847 | 84888 | 53963 | 14060 | 2390 | 173148 |

### Table S2 | Sensitivity analysis of clustering stability across optimal parameters.

Table S2: **Sensitivity analysis of clustering stability across optimal parameters.** This table displays the assigned cluster ID for each country from the k-means clustering, performed using the optimal number of clusters (k) for each tested number of top lineages (N). The purpose of this analysis is to demonstrate the robustness of the identified epidemiological basins.

|  | **cluster_id_N25_k6** | **cluster_id_N30_k7** | **cluster_id_N40_k7** | **cluster_id_N50_k7** |
| --- | --- | --- | --- | --- |
| **country** |  |  |  |  |
| **Algeria** | 5 | 6 | 5 | 2 |
| **Angola** | 4 | 3 | 2 | 3 |
| **Benin** | 5 | 0 | 0 | 0 |
| **Botswana** | 1 | 1 | 2 | 3 |
| **Burkina Faso** | 5 | 6 | 5 | 2 |
| **Burundi** | 1 | 1 | 5 | 2 |
| **Cabo Verde** | 5 | 2 | 4 | 5 |
| **Cameroon** | 5 | 6 | 5 | 2 |
| **Central African Republic** | 5 | 6 | 5 | 2 |
| **Chad** | 2 | 0 | 0 | 0 |
| **Comoros** | 3 | 5 | 6 | 6 |
| **Cote d’Ivoire** | 5 | 6 | 5 | 2 |
| **Democratic Republic of the Congo** | 5 | 1 | 5 | 2 |
| **Djibouti** | 4 | 6 | 5 | 2 |
| **Egypt** | 5 | 6 | 5 | 2 |
| **Equatorial Guinea** | 4 | 3 | 2 | 3 |
| **Eswatini** | 4 | 3 | 2 | 3 |
| **Ethiopia** | 5 | 6 | 5 | 2 |
| **Gabon** | 5 | 6 | 5 | 2 |
| **Gambia** | 5 | 2 | 4 | 5 |
| **Ghana** | 5 | 6 | 5 | 2 |
| **Guinea** | 5 | 6 | 5 | 2 |
| **Guinea-Bissau** | 5 | 6 | 5 | 2 |
| **Kenya** | 5 | 1 | 5 | 2 |
| **Lesotho** | 4 | 3 | 2 | 3 |
| **Liberia** | 5 | 6 | 5 | 0 |
| **Libya** | 5 | 0 | 0 | 0 |
| **Madagascar** | 3 | 5 | 6 | 6 |
| **Malawi** | 4 | 3 | 2 | 3 |
| **Mali** | 5 | 6 | 5 | 2 |
| **Mauritania** | 5 | 2 | 4 | 5 |
| **Mauritius** | 5 | 6 | 1 | 1 |
| **Mayotte** | 3 | 5 | 6 | 6 |
| **Morocco** | 5 | 6 | 5 | 2 |
| **Mozambique** | 4 | 3 | 2 | 3 |
| **Namibia** | 1 | 1 | 2 | 3 |
| **Niger** | 5 | 6 | 5 | 2 |
| **Nigeria** | 2 | 0 | 0 | 0 |
| **Republic of the Congo** | 5 | 6 | 5 | 2 |
| **Reunion** | 3 | 5 | 6 | 6 |
| **Rwanda** | 4 | 1 | 5 | 2 |
| **Sao Tome and Principe** | 5 | 6 | 5 | 2 |
| **Senegal** | 5 | 2 | 4 | 5 |
| **Seychelles** | 0 | 4 | 3 | 4 |
| **Sierra Leone** | 5 | 6 | 5 | 2 |
| **Somalia** | 5 | 6 | 5 | 2 |
| **South Africa** | 4 | 3 | 2 | 3 |
| **South Sudan** | 1 | 1 | 5 | 2 |
| **Sudan** | 5 | 6 | 5 | 2 |
| **Tanzania** | 4 | 3 | 2 | 3 |
| **Togo** | 0 | 4 | 3 | 4 |
| **Tunisia** | 0 | 4 | 3 | 4 |
| **Uganda** | 1 | 1 | 5 | 2 |
| **Zambia** | 4 | 3 | 2 | 3 |
| **Zimbabwe** | 4 | 3 | 2 | 3 |

### Table S3 | Annual and Cumulative SARS-CoV-2 Cases and Deaths for the Africa Study Cohort.

(Table S3 was provided separately in Excel format in the supplementary files folder.)

This table delineates the epidemiological burden of SARS-CoV-2 across all African countries included in our genomic analysis (the "study cohort"). Data are aggregated to show the total number of reported cases and deaths for each calendar year, providing a clear view of the pandemic's temporal waves within our specific cohort. Cumulative totals for the entire period are provided for each country, allowing for direct comparison of the overall pandemic impact. The 'Total' row summarizes these metrics across all cohort countries, serving as a foundational epidemiological reference for the spatiotemporal and genomic analyses presented in the main manuscript.

### Table S4 | Pre-Omicron lineage-country contingency matrix.

(Table S4 was provided separately in Excel format in the supplementary files folder.)

The table displays the raw counts of genomic sequences for the 30 most frequent SARS-CoV-2 lineages (columns) identified across African nations (rows) during the pre-Omicron era (2020-2021). The values represent the absolute frequency of each lineage within each country in the curated dataset. This matrix formed the primary quantitative input for the Correspondence Analysis (CA) and subsequent hierarchical clustering used to define the seven distinct epidemiological basins described in the main text. Total counts for each country (row totals) and each lineage (column totals) are included for comprehensive reference.

### Table S5 | Characteristics and virological signatures of the seven epidemiological basins.

Tableau S5: The table provides a detailed summary for each of the seven clusters identified in the study. Clusters were defined via K-means clustering of country coordinates derived from a Correspondence Analysis of the pre-Omicron (2020-2021) lineage contingency matrix (Table S2). For each cluster, the table lists its descriptive name, the total number of member countries, and a comprehensive list of these nations. The final three columns detail the unique virological signature of each basin by identifying the top three most abundant lineages. The percentage in parentheses indicates the lineage's relative frequency among all top-30 lineage sequences within that specific cluster, highlighting the primary drivers of the regional grouping.

| **Cluster ID** | **Cluster Name** | **Size** | **Member Countries** | **Top Lineage 1** | **Top Lineage 2** | **Top Lineage 3** |
| --- | --- | --- | --- | --- | --- | --- |
| **0** | West/Central Africa - Eta/Delta Mix | 4 | Benin, Chad, Libya, Nigeria | AY.36 (35.7%) | BA.1.1 (20.1%) | B.1.525 (11.8%) |
| **1** | East/Southern Africa - Diverse Delta Waves | 8 | Botswana, Burundi, Democratic Republic of the Congo, Kenya, Namibia, Rwanda, South Sudan, Uganda | B.1 (15.8%) | AY.46 (15.2%) | BA.1.1 (14.6%) |
| **2** | Atlantic West Africa - Early Pandemic Waves | 4 | Cabo Verde, Gambia, Mauritania, Senegal | B.1 (26.7%) | AY.34.1 (12.1%) | B.1.1.420 (11.3%) |
| **3** | Southern Africa - Beta & Early Omicron Epicenter | 10 | Angola, Equatorial Guinea, Eswatini, Lesotho, Malawi, Mozambique, South Africa, Tanzania, Zambia, Zimbabwe | B.1.351 (30.0%) | AY.45 (18.5%) | BA.1 (12.5%) |
| **4** | Disparate - Alpha & Specific Delta Wave | 3 | Seychelles, Togo, Tunisia | AY.122 (54.3%) | B.1.1.7 (19.7%) | B.1.617.2 (6.2%) |
| **5** | Indian Ocean Islands - Beta/Delta Introduction | 4 | Comoros, Madagascar, Mayotte, Reunion | B.1.351.2 (28.5%) | AY.40 (21.2%) | AY.43 (18.9%) |
| **6** | Pan-African - Generic Delta Wave | 22 | Algeria, Burkina Faso, Cameroon, Central African Republic, Cote d’Ivoire, Djibouti, Egypt, Ethiopia, Gabon, Ghana, Guinea, Guinea-Bissau, Liberia, Mali, Mauritius, Morocco, Niger, Republic of the Congo, Sao Tome and Principe, Sierra Leone, Somalia, Sudan | B.1.617.2 (21.9%) | B.1 (16.6%) | B.1.1.7 (14.3%) |

### **Table S6 | Distribution of major SARS-CoV-2 clades across the seven epidemiological basins.**

Tableau S6: The table presents the absolute counts of high-quality genomic sequences for each major SARS-CoV-2 clade (columns) across the seven epidemiological basins (rows) defined in the study. The values represent the total number of sequences assigned to a specific clade within a given cluster over the entire pandemic period analyzed. This provides a comprehensive overview of the relative impact of successive pandemic waves on each distinct geographical region. The 'Total' column quantifies the total number of sequences from each cluster included in this analysis, while the 'Total' row provides the continent-wide sequence count for each clade.

|  | **G** | **GH** | **GK** | **GR** | **GRA** | **GRY** | **GV** | **L** | **O** | **S** | **V** | **Total** |
| --- | --- | --- | --- | --- | --- | --- | --- | --- | --- | --- | --- | --- |
| **West/Central Africa - Eta/Delta Mix** | 1141 | 121 | 3352 | 787 | 3989 | 308 | 9 | 36 | 63 | 67 | 9 | 9882 |
| **East/Southern Africa - Diverse Delta Waves** | 2262 | 2020 | 5880 | 803 | 11798 | 1044 | 11 | 6 | 99 | 632 | 9 | 24564 |
| **Atlantic West Africa - Early Pandemic Waves** | 2468 | 264 | 1668 | 1257 | 2935 | 304 | 15 | 25 | 25 | 63 | 1 | 9025 |
| **Southern Africa - Beta & Early Omicron Epicenter** | 3070 | 10139 | 15621 | 5312 | 31722 | 381 | 193 | 8 | 111 | 32 | 8 | 66597 |
| **Disparate - Alpha & Specific Delta Wave** | 283 | 317 | 2000 | 246 | 1829 | 573 | 66 | 0 | 17 | 65 | 1 | 5397 |
| **Indian Ocean Islands - Beta/Delta Introduction** | 210 | 4330 | 5910 | 390 | 12707 | 181 | 168 | 0 | 17 | 15 | 0 | 23928 |
| **Pan-African - Generic Delta Wave** | 2505 | 1567 | 4614 | 3186 | 19441 | 1018 | 44 | 41 | 658 | 652 | 4 | 33730 |
| **Total** | 11939 | 18758 | 39045 | 11981 | 84421 | 3809 | 506 | 116 | 990 | 1526 | 32 | 173123 |

### Table S7 | Significant Spearman correlations between lineage prevalence and epidemiological indicators.

(Table S7 was provided separately in Excel format in the supplementary files folder.)

The table presents a curated summary of all statistically significant (p < 0.05) correlations identified between the monthly prevalence of SARS-CoV-2 lineages and the smoothed counts of new cases and new deaths within each epidemiological basin. For each lineage, the corresponding clade and variant name are provided. The analysis employed a time-lagged Spearman's rank correlation, testing lags of 0, 1, and 2 months to identify the strongest association. The reported Spearman's Rho (ρ) represents the coefficient at the optimal time lag, which is also indicated. 95% Confidence Intervals (CIs) were estimated using a non-parametric bootstrap procedure (1,000 iterations).

### Table S8 | Summary of Spearman correlations between clade prevalence and epidemiological indicators.

(Table S8 was provided separately in Excel format in the supplementary files folder.)

The table presents the complete results of the time-lagged Spearman's rank correlation analysis between the monthly prevalence of major SARS-CoV-2 clades and the smoothed counts of new cases and new deaths within each epidemiological basin. The analysis tested time lags of 0, 1, and 2 months and reports the Spearman's Rho (ρ) for the strongest significant correlation (p < 0.05). A value of 0 indicates that no statistically significant correlation was found at any of the tested lags. The corresponding 95% Confidence Intervals (CIs), estimated via 1,000 bootstrap iterations, are provided for all significant results. This table provides a comprehensive macro-level view of the relationship between large-scale viral waves and their epidemiological impact across the continent.

### Table S9 | Complete results of the cluster-specific Ridge Regression models.

(Table S9 was provided separately in Excel format in the supplementary files folder.)

The table presents the comprehensive results from the multivariate Ridge Regression models fitted for each epidemiological basin. The models predict monthly deaths based on the monthly prevalence of co-circulating SARS-CoV-2 clades and the mean vaccination rate. To ensure model stability and robustness, a stringent filtering procedure was applied: only clades with a peak prevalence exceeding 3% and a mean prevalence exceeding 1% within a given cluster were included as predictors. All predictors were standardized before model fitting. The columns are defined as follows:

• Cluster Name: The epidemiological basin for which the model was built.

• Predictor Variable: The independent variable (clade or vaccination rate) used in the model.

• Standardized Coefficient: The regression coefficient, representing the change in monthly deaths for a one-standard-deviation increase in the predictor, holding all other variables constant.

• Status: Indicates whether the predictor was 'Included in model' or 'Excluded from model (low prevalence)' based on the filtering criteria.

• Model R-squared: The coefficient of determination for the entire model, indicating the proportion of variance in monthly deaths explained by the predictors.

• Model Best Alpha: The optimal regularization parameter (α) selected by the leave-one-out cross-validation procedure within RidgeCV, which controls the degree of coefficient shrinkage to prevent overfitting.

### Table S10 | Sensitivity analysis of the time-lagged Case Fatality Rate (CFR).

(Table S10 was provided separately in Excel format in the supplementary files folder.)

The table presents the complete results of the time-lagged Case Fatality Rate (CFR) analysis, calculated with a one-month lag between case and death reporting (deaths in month M / cases in month M-1). To ensure the robustness of our findings, the analysis was conducted across three different thresholds of clade dominance (50%, 60%, and 75%), and the results for each are presented. For each calculation, the table provides the resulting CFR, the 95% confidence interval calculated using the Wilson score interval method, and the absolute number of smoothed cases and deaths used as the denominator and numerator, respectively.

### Table S11 | Complete results of the multivariate logistic regression analysis of demographic risk factors.

The table presents the comprehensive results of the multivariate logistic regression models fitted for each major SARS-CoV-2 clade. For each clade, a separate model was fitted to identify demographic predictors of infection, controlling for both age and sex. The outcome variable was infection with the specific clade of interest. The table details the Adjusted Odds Ratios (aOR) and their corresponding 95% Confidence Intervals (CIs). An aOR greater than 1 indicates an increased risk of infection for that demographic group compared to the reference group, while an aOR less than 1 indicates a decreased risk. The reference categories used were 'Female' for sex and 'Adults (30-49 years)' for age. These statistical results underpin the demographic risk profiles visualized in **Figure 6**.

| **Clade (Variant)** | **Predictor Variable** | **Adjusted OR** | **95% Confidence Interval** |
| --- | --- | --- | --- |
| **G** | age_child (0-9) | 0.52 | [0.45, 0.59] |
| **G** | age_old (70+) | 0.69 | [0.63, 0.76] |
| **G** | age_older_adult (50-69) | 0.89 | [0.84, 0.93] |
| **G** | age_teenage (10-17) | 0.64 | [0.58, 0.70] |
| **G** | age_young_adult (18-29) | 0.99 | [0.94, 1.04] |
| **G** | sex_Male | 1.39 | [1.34, 1.45] |
| **GH (Beta)** | age_child (0-9) | 0.91 | [0.83, 1.00] |
| **GH (Beta)** | age_old (70+) | 0.88 | [0.82, 0.95] |
| **GH (Beta)** | age_older_adult (50-69) | 1.06 | [1.02, 1.11] |
| **GH (Beta)** | age_teenage (10-17) | 1.11 | [1.04, 1.19] |
| **GH (Beta)** | age_young_adult (18-29) | 1.08 | [1.03, 1.13] |
| **GH (Beta)** | sex_Male | 1 | [0.96, 1.03] |
| **GK (Delta)** | age_child (0-9) | 1.13 | [1.06, 1.20] |
| **GK (Delta)** | age_old (70+) | 0.83 | [0.79, 0.87] |
| **GK (Delta)** | age_older_adult (50-69) | 0.98 | [0.95, 1.01] |
| **GK (Delta)** | age_teenage (10-17) | 1.28 | [1.22, 1.35] |
| **GK (Delta)** | age_young_adult (18-29) | 1.1 | [1.06, 1.13] |
| **GK (Delta)** | sex_Male | 0.96 | [0.94, 0.99] |
| **GR (Gamma)** | age_child (0-9) | 0.67 | [0.59, 0.76] |
| **GR (Gamma)** | age_old (70+) | 0.8 | [0.73, 0.87] |
| **GR (Gamma)** | age_older_adult (50-69) | 0.92 | [0.87, 0.97] |
| **GR (Gamma)** | age_teenage (10-17) | 0.71 | [0.65, 0.79] |
| **GR (Gamma)** | age_young_adult (18-29) | 0.93 | [0.88, 0.99] |
| **GR (Gamma)** | sex_Male | 1.05 | [1.00, 1.09] |
| **GRA (Omicron)** | age_child (0-9) | 1.26 | [1.20, 1.33] |
| **GRA (Omicron)** | age_old (70+) | 1.39 | [1.33, 1.45] |
| **GRA (Omicron)** | age_older_adult (50-69) | 1.05 | [1.02, 1.08] |
| **GRA (Omicron)** | age_teenage (10-17) | 0.99 | [0.95, 1.03] |
| **GRA (Omicron)** | age_young_adult (18-29) | 0.94 | [0.91, 0.97] |
| **GRA (Omicron)** | sex_Male | 0.9 | [0.88, 0.92] |
| **GRY (Alpha)** | age_child (0-9) | 0.66 | [0.53, 0.82] |
| **GRY (Alpha)** | age_old (70+) | 0.91 | [0.79, 1.06] |
| **GRY (Alpha)** | age_older_adult (50-69) | 1.03 | [0.94, 1.13] |
| **GRY (Alpha)** | age_teenage (10-17) | 0.83 | [0.71, 0.97] |
| **GRY (Alpha)** | age_young_adult (18-29) | 0.91 | [0.83, 1.01] |
| **GRY (Alpha)** | sex_Male | 1.21 | [1.13, 1.30] |
| **GV** | age_child (0-9) | 0.98 | [0.60, 1.62] |
| **GV** | age_old (70+) | 1.02 | [0.70, 1.49] |
| **GV** | age_older_adult (50-69) | 1.11 | [0.87, 1.42] |
| **GV** | age_teenage (10-17) | 1.45 | [1.03, 2.04] |
| **GV** | age_young_adult (18-29) | 0.91 | [0.70, 1.19] |
| **GV** | sex_Male | 0.89 | [0.74, 1.07] |
| **O** | age_child (0-9) | 0.42 | [0.24, 0.73] |
| **O** | age_old (70+) | 0.67 | [0.48, 0.95] |
| **O** | age_older_adult (50-69) | 0.67 | [0.54, 0.84] |
| **O** | age_teenage (10-17) | 0.63 | [0.44, 0.91] |
| **O** | age_young_adult (18-29) | 0.8 | [0.64, 0.98] |
| **O** | sex_Male | 1.44 | [1.22, 1.69] |
| **S** | age_child (0-9) | 0.4 | [0.25, 0.63] |
| **S** | age_old (70+) | 0.74 | [0.57, 0.97] |
| **S** | age_older_adult (50-69) | 0.88 | [0.75, 1.03] |
| **S** | age_teenage (10-17) | 0.51 | [0.36, 0.70] |
| **S** | age_young_adult (18-29) | 1.13 | [0.97, 1.32] |
| **S** | sex_Male | 1.85 | [1.63, 2.10] |

### Table S12 | Comprehensive Z-score analysis of demographic tropism by clade.

(Table S12 was provided separately in Excel format in the supplementary files folder.)

The table details the results of the Z-score analysis used to quantify the over- or under-representation of specific demographic groups (defined by age and gender) within each SARS-CoV-2 clade. This analysis complements the logistic regression by measuring the deviation of a group's prevalence within a specific clade (p1​) compared to its prevalence in the remainder of the dataset (p2​). A positive Z-score indicates significant over-representation (specific tropism), while a negative Z-score indicates under-representation (protective effect).

### Table S13 | Comprehensive summary of the major Nextstrain clades observed in Africa during 2020-2024 period.

**Tableau S13** : This table provides a comprehensive summary of the major Nextstrain clades observed in Africa during the study period. For each clade, it lists the corresponding WHO variant label, the primary Pangolin lineage that defines the variant, and other notable lineages identified through a systematic screen combining continental prevalence, regional dominance, and significant epidemiological impact (see Methods). This provides a reference map for the key viral actors of the pandemic in Africa as revealed by our study.

| Clade (Nextstrain) | Variant (WHO Label) | Defining Pango Lineage | Other Notable Pango Lineages Identified in Africa | Key Narrative Role in Our Study |
| --- | --- | --- | --- | --- |
| **G** | (Ancestral) | B.1 | B, B.1.416, B.1.525, B.1.620 | Formed the initial pandemic wave; associated with the highest "situational lethality" (CFR) and a strong tropism for adult males. |
| **S** | (Ancestral) | A | A.23.1, A.27 | Represents an early, distinct ancestral branch with a notable footprint in East Africa. |
| **GRY** | **Alpha** | B.1.1.7 | - | Drove the first major continental replacement wave, marking the transition to Variants of Concern. |
| **GH** | **Beta** | **B.1.351** | AY.19, B.1.160, B.1.351.2 | The archetypal regional variant, defining the "Southern Africa" epidemiological basin and its unique epidemic trajectory. |
| **GR** | **Gamma & others** | B.1.1 | AZ.5, B.1.1.1, B.1.1.318, B.1.1.420, B.1.1.448, B.1.1.54, C.1, C.1.2, C.17 | A diverse group of early lineages showing complex, region-dependent impacts on mortality. |
| **GV** | (N/A) | B.1.177 | - | An early European lineage with limited but detectable circulation on the continent. |
| **GK** | **Delta** | B.1.617.2 | **AY.45 (Southern Africa), AY.34.1 (West Africa), AY.122 (Disparate), AY.16, AY.36, AY.40, AY.43, AY.46, and numerous others.** | Identified as the primary driver of mortality (highest relative impact). Our analysis revealed its seemingly monolithic wave was in fact a "mosaic" of distinct regional epidemics driven by highly successful sub-lineages. |
| **GRA** | **Omicron** | B.1.1.529 | BA.1, BA.1.1, BA.2, BA.4, BA.4.1, BA.5.3.1, BQ.1.1, BF.5, and multiple BA.1.1 sub-lineages. | Caused a fundamental decoupling of cases from mortality. Drove the third demographic regime, a "U-shaped" risk profile targeting the very young and elderly. |

## Supplementary Figures:

### Figure S1 | Defining lineage-based epidemiological clusters in Africa using Correspondence Analysis.

**

**

**Figure S1:** This figure details the methodology and results of the Correspondence Analysis (CA) used to partition African nations into seven cohesive epidemiological clusters based on their pre-Omicron (2020-2021) SARS-CoV-2 lineage profiles. (A) Scree plot of explained inertia. The bar chart (blue, left y-axis) shows the percentage of the total inertia (variance) in the lineage circulation data explained by each of the first 20 CA components. The line plot (red, right y-axis) shows the cumulative inertia, indicating that the first five components capture over 62% of the total variance, justifying their use for clustering. (B) Elbow method for optimal k-cluster selection. The plot shows the within-cluster sum of squares (inertia) as a function of the number of clusters (k), calculated using the K-Means algorithm on the coordinates of the first five CA components. The "elbow point," annotated at k=7, represents the optimal trade-off between maximizing the number of clusters and minimizing the variance within each cluster. (C) CA biplot of country and lineage profiles. This plot visualizes the relationships between countries (points) and the dominant Pango lineages (red text) along the first two dimensions of the CA. Countries are colored according to their final cluster assignment (k=7). Proximity between a country and a lineage indicates a higher relative prevalence of that lineage in that country during the pre-Omicron period.

### Figure S2 | Comparative clustering analysis on the full pandemic period reveals the homogenizing effect of Omicron.





**Figure S2:** This figure details the methodology and results of a comparative clustering analysis performed on the entire 2020-2024 dataset, based on the 30 most frequent Pango lineages of this period. This analysis serves to validate the choice of the pre-Omicron period for the main analysis presented in the article by demonstrating the structural impact of the Omicron waves on continental clustering. (A) Scree plot of the eigenvalues from the Correspondence Analysis (CA). Bars represent the percentage of total inertia (variance) explained by each principal component. The red line indicates the cumulative explained inertia. (B) Elbow method plot used to determine the optimal number of clusters (k). The plot shows the within-cluster sum of squares (inertia) as a function of k. The "elbow point," highlighted in red at k=7, represents the point of diminishing returns where adding more clusters provides progressively smaller gains in explained variance, indicating the optimal balance between model complexity and explanatory power. (C) Symmetrical biplot of the first two dimensions of the CA. Points represent African countries, colored according to their cluster assignment based on the full-period analysis. The proximity of countries indicates similarity in their viral lineage profiles over the entire pandemic. Dominant Pango lineages are shown in red, positioned at the weighted average of the countries they most characterize. This visualization highlights the formation of a large, central "Pan-African" cluster (green), reflecting the widespread, homogenizing distribution of Omicron sublineages in the later period of the pandemic, in contrast to the more distinct regional clusters of the pre-Omicron era.

### Figure S3 | Systematic validation of clustering parameters.

**

**

**Figure S3:** To justify the selection of the number of top lineages (N) and the number of clusters (k), a systematic evaluation was performed using three objective metrics. Each colored line represents a different value of N, as detailed in the legend panel. Red circles indicate the optimal k identified for each N and metric. (A) The normalized elbow method plot shows the clearest inflection point at k=7 for N=30. (B) The silhouette score, which measures cluster separation, consistently peaks at k=7 for all N ≥ 30, providing strong evidence for a robust seven-cluster structure. (C) The Calinski-Harabasz score. Taken together, these metrics demonstrate a strong consensus for (N=30, k=7) as the optimal and most parsimonious choice that reveals a clear and stable epidemiological structure in the data

### Figure S4 | The dominance of B.1 at continental level and the diversity of circulating lineages in early stages of SARS-CoV-2 pandemic.

**

**

**Figure S4:** A map of Africa showing countries coloured by their dominant lineage in 2020. The second part shows the proportion of lineages characterising genomes sequenced in each country. The five most prevalent lineages are displayed individually, while all others are categorised under the label 'Others' (in black).

### Figure S5 | Clusters clade evolving distribution in our lineage-based clustering study.





**Figure S5**: The figure presents a spatiotemporal analysis of SARS-CoV-2 clade dynamics. (A), (C), (D), (E), (F) Annual clade composition for each of the seven epidemiological clusters from 2020 to 2024. Each horizontal bar chart represents a distinct cluster. Segments within each bar show the relative proportion of the top five most frequent clades identified for that specific cluster and year. All other detected clades are aggregated into the 'Others' category (black). The color of the text overlaying each segment is dynamically chosen for maximal contrast and readability. (B) Geographic distribution of the seven epidemiological clusters. These clusters were defined based on viral lineage circulation patterns during the 2020-2021 pre-Omicron period. Each country is colored according to its cluster assignment, providing a visual representation of the distinct epidemiological regions identified through our analysis.

### Figure S6 | Omicron (GRA clade) phylogenetic tree.

**
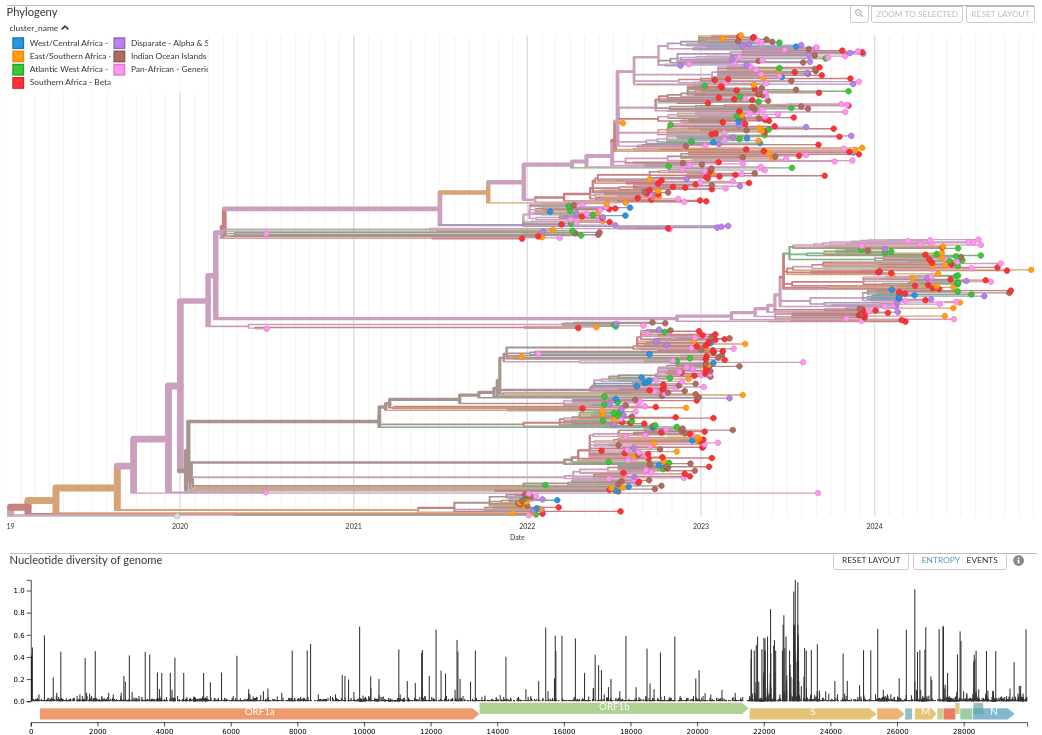
**

**Figure S6:** (a) A time-calibrated phylogeny of 603 sequences from the GRA (Omicron) clade, sampled to represent the genetic diversity across seven predefined African epidemiological clusters. The tree layout and x-axis are consistent with Fig. 1. Branches are colored by the epidemiological cluster of origin, as defined in our previous ecological analysis (see Methods), revealing the introduction and subsequent expansion of Omicron lineages within specific regions. (b) The lower panel shows the genomic diversity within the Omicron alignment. This detailed view highlights multiple independent introductions of Omicron sub-lineages into different African regions and their subsequent local diversification

### Figure S7 | Optimal time-lagged correlations between SARS-CoV-2 clade prevalence and epidemiological impact across African clusters.

**

**

**Figure S7:** The heatmaps display the optimal and statistically significant (Spearman's ρ, p < 0.05) temporal correlation between the monthly proportion of major SARS-CoV-2 clades and the subsequent waves of (a) new cases and (b) new deaths. Rows represent the seven pre-defined epidemiological clusters. Columns represent major GISAID clades, labeled with their corresponding WHO variant name. Cell color intensity indicates the strength and direction of the Spearman correlation coefficient (ρ), from strong negative correlation (blue) to strong positive correlation (red). Cell annotations display the optimal correlation coefficient, with its corresponding 95% confidence interval—calculated via a 1,000-iteration bootstrap procedure—shown below it to indicate the estimate's robustness. White cells indicate that no statistically significant and robust correlation was found for that clade-cluster pair.

### Figure S8 | Variant-specific demographic profiles reveal a shift in age tropism during the SARS-CoV-2 pandemic in Africa.

**
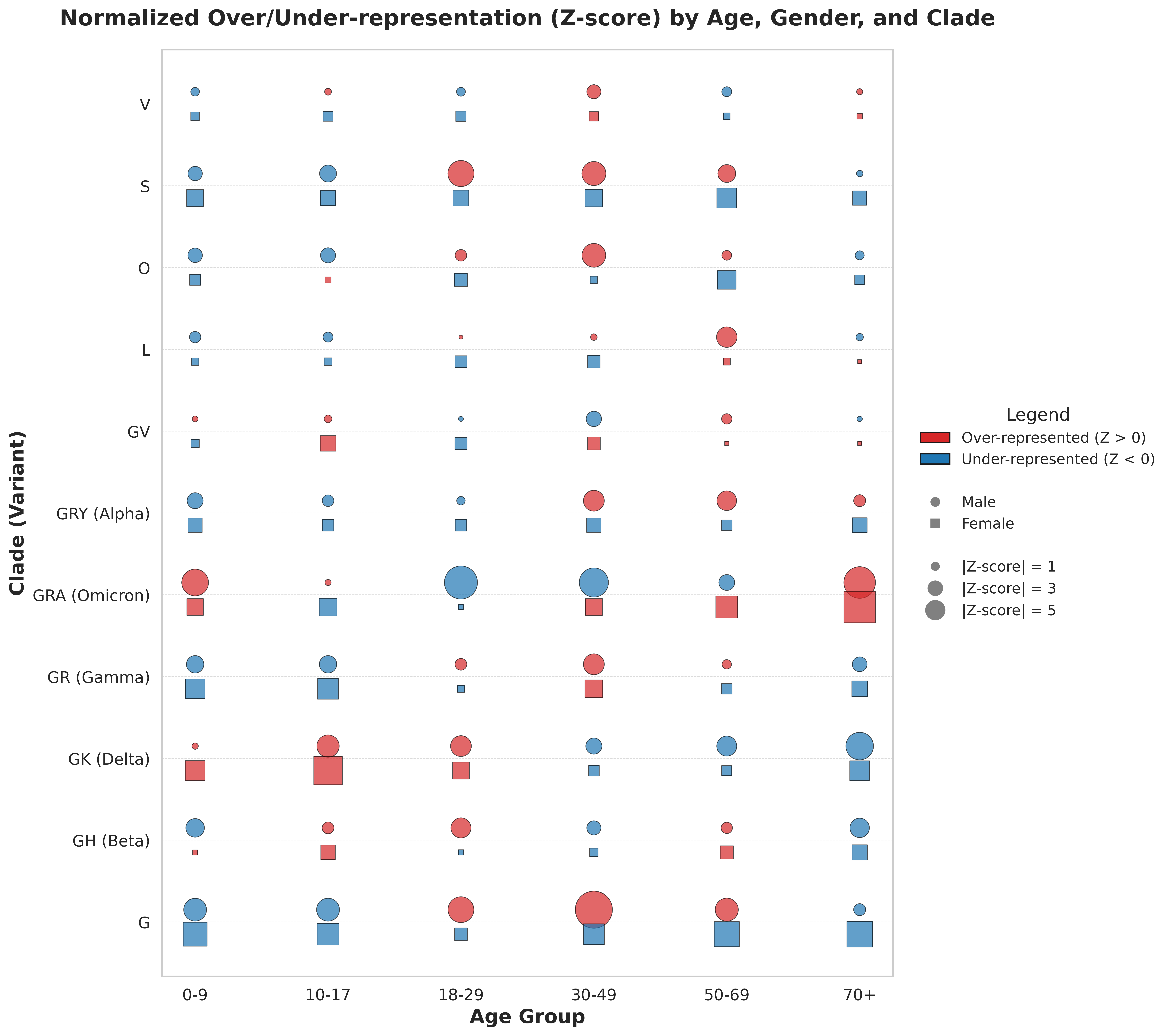
**

**Figure S8:** The bubble plot displays the Z-scores for SARS-CoV-2 clades across six age groups and two genders. This analysis visualizes the relative over-representation (red, Z-score > 0) or under-representation (blue, Z-score < 0) of each clade within a specific demographic stratum. The size of each bubble is directly proportional to the absolute value of the Z-score, highlighting the magnitude of the deviation from the expected frequency. The expected frequency is derived from the baseline demographic distribution of the entire sequenced cohort (n = 149148 samples). Circles represent male individuals, and squares represent female individuals, with a slight vertical offset for visual clarity. The plot reveals a distinct age-related signature for major variants, notably a tropism for younger populations in Delta (GK) and a bimodal tropism for the very young and the elderly in Omicron (GRA), contrasting sharply with the male-dominant, middle-aged profile of ancestral lineages (e.g., G).
